# Supplementary material for: Genome-wide identification and characterization of TCP family genes in Brassica juncea var. tumida
Source: PeerJ. 2020 May 14;8:e9130. doi: 10.7717/peerj.9130 (PMC7231505; doi:10.7717/peerj.9130)
Supplement: Figure S1 [file peerj-08-9130-s001.pdf]

```
                                                                  1
BjTCP19   .......................................................MESNL
AtTCP19   .......................................................MESNH
BjTCP9a   .....................................................MATIKKH
BjTCP9c   .....................................................MATIKTH
BjTCP9b   .....................................................MATIKHH
BjTCP9d   .....................................................MATIKKH
AtTCP9    .....................................................MATIQKL
AtTCP11   ............................................................
BjTCP21b  ............................................................
BjTCP21d  ............................................................
BjTCP21a  ............................................................
BjTCP21e  ............................................................
BjTCP21c  ............................................................
BjTCP21f  ............................................................
AtTCP21   ............................................................
BjTCP7a   ............................................................
BjTCP7e   ............................................................
BjTCP7b   ............................................................
BjTCP7d   ............................................................
BjTCP7c   ............................................................
AtTCP7    ............................................................
BjTCP22a  ............................................................
BjTCP22c  ............................................................
AtTCP22   ............................................................
BjTCP22b  ............................................................
BjTCP22d  ............................................................
AtTCP23   ............................................................
BjTCP8a   ............................................................
BjTCP8b   ............................................................
AtTCP8    ............................................................
BjTCP15a  ............................................................
BjTCP15d  ............................................................
BjTCP15c  ............................................................
BjTCP15e  ............................................................
BjTCP15b  ............................................................
BjTCP15f  ............................................................
AtTCP15   ............................................................
BjTCP14a  ............................................................
BjTCP14b  ................................................MDGGDNVGGGGG
AtTCP14   ......................................MQKPTSSILNVIMDGGDSVGGG
BjTCP20a  ............................................................
BjTCP20c  ............................................................
AtTCP20   ............................................................
BjTCP20b  ............................................................
BjTCP6    ............................................................
AtTCP6    ............................................................
BjTCP1a   ......MSSSNNDYNNG.NSGVYPLSLYLSSLPGHQGIIRNPYN................
BjTCP1e   .........SNNDYSNG.NNGVYPLSLYLSSLPGQQGIICNPYN................
BjTCP1b   ......MSSSNNDYING.NNGVYPLSLYLSPLAGHQDIIRNPYN................
BjTCP1c   ......MSSFNNDYNNG.NNGVYPLSLYLSPLAGHQDINRNPYN................
BjTCP1d   ......MSSYNNNYNNGSNNGVYPLSLYLSSLSGHQDIIRNPYN................
AtTCP1    ......MSSSTNDYNDGNNNGVYPLSLYLSSLSGHQDIIHNPYN................
BjTCP12a  ........MFPSIDTNGYDLFDPFNLHEATMLPSFTTHILSPSS................
BjTCP12b  ........MFPSIDTNGYDLFDPFNLHEATLLPSFTTHIQCPSS................
AtTCP12   ........MFPSLDTNGYDLFDPFIPHQTTMFPSFITHIQSPNS................
BjTCP18a  .......MNNRSFSTTTTISEDYMLFPYNDHYSSQPLLPFNPCSSINDILIHSNSNISSN
BjTCP18d  ......MNDNKTFSTTTTINDDYMLFPYDDHYSSQPLLPFSPCSSINDILIHSNSNTTNN
BjTCP18b  ......MNNNKTFSTTSTINEDYMSFPYNDNYCSQPLLPFSPSSSINDLLIHSNSNTSNN
BjTCP18c  ......MNNNKTFSTTSTINEDYMSFPYNDNYCSQPLLPFSPSSSINDLLIHSNSNTSNN
AtTCP18   .......MNNNIFSTTTTINDDYMLFPYNDHYSSQPLLPFSPSSSINDILIHSTSNTSNN
BjTCP17a  ............................................................
BjTCP17b  ......................................MRTN..................
AtTCP17   ............................................................
BjTCP5c   ............MRSREYDDEETQAKQERDHN.HNNQVNLNN..................
BjTCP5a   .........................................M..................
BjTCP5b   ............MRSKECDEEEIQAKQEGDHNDNHNHQVNLN..................
AtTCP5    ............MRSGECDEEEIQAKQERDQ..NQNHQVNLN..................
BjTCP13a  ............MDTGPWKDTNDDVSGQTRTRREGESE......................
BjTCP13c  ............MDTVHWKDTNDDVSGQTRTR.HEEAV......................
BjTCP13b  MTVQTQELNTSIMNTGPWRDANDDVSGGTRTRRNGEVEEDSEEA................
AtTCP13   ............MNIVSWKDANDEVAGGATTRREREVKEDQE..................
BjTCP24a  .......................................MEVDD................
BjTCP24b  .......................................MEVDD................
BjTCP24c  ....................MEDDDHTETQQQQQQQQQQQQQQQ................
BjTCP24d  .............................EKHESSVSMEEDDDI................
AtTCP24   .....................................MEVDEDI................
BjTCP2a   ............................................................
BjTCP2d   ............................................................
BjTCP2b   ............................................................
BjTCP2c   ............................................................
AtTCP2    ............................................................
BjTCP3    ............................................................
AtTCP3    .......................................MAPDN................
AtTCP4    ...........................................M................
AtTCP16   ............................................................
AtTCP10   ............................................................
```

```
                          10        20        30
BjTCP19   EG.............KEAMAHLSDPDPETNRGMLLKQEDDDPNKQ...............
AtTCP19   EGNA.....IQVIDQVTTMTHLSDPNPKTKPGMMLMKQEDG...................
BjTCP9a   EAER.....LRAVDLSIIDGVRSVETSKPFQVANTVSLDPKAEPL...............
BjTCP9c   EAER.....LRAVDPSLINGVRSVETSKPSEVANTVSLEPKAEPS...............
BjTCP9b   EDER.....LRAVDLTSKP.....FEVNN.AVSLEPKTEPSLESS...............
BjTCP9d   EGER.....LRAVDLSVQT.....FEVNNNAVSLEPKTEPSSLES...............
AtTCP9    EEVAGKDQTLRAVDLTIINGVRNVETSRPFQVNPTVSLEPKAEP................
AtTCP11   ....................MIFQNVCRNESNFNAIASESRSQTQ...............
BjTCP21b  ..................................MENSDGA...................
BjTCP21d  ..................................MENNDGA...................
BjTCP21a  ..................................MTSNDGS...................
BjTCP21e  .................................MTSNDGGG...................
BjTCP21c  ..................................MSNDDGT...................
BjTCP21f  ..................................MSNDDGT...................
AtTCP21   ..................................MADNDGA...................
BjTCP7a   ........................MSNNDGVMISNGSLIEH...................
BjTCP7e   .......................MSNNNDGVMISNGSLMEH...................
BjTCP7b   ............................................................
BjTCP7d   ............................................................
BjTCP7c   ............................................................
AtTCP7    .............................MSINNNNNNNNN...................
BjTCP22a  .................MDQNSHDR.PTAEAGLQLNSTENPSPGRSVHFAGG........
BjTCP22c  .................MDQNSHDRPAAAEAGLQLNSTENPSPGRSVPFAS.........
AtTCP22   .....................MNQNSSVAEATLQLNSGEKPSPGSIPFISSG........
BjTCP22b  ......................METRTAEEASLQLNSTENPSPGG...............
BjTCP22d  .....................METRTAEAEASLQLNSTENQSPGG...............
AtTCP23   ................MESHNNNQSNNNTTGSAHLVPSMGPISGSVS.............
BjTCP8a   ...MDLSDD........GAAARGGSRHLVDPSLSIVPRSTP...................
BjTCP8b   ...MDLSDD........GAAARGSSRHLVDPSLSIVPRSTP...................
AtTCP8    ...MDLSDIRNNNNDTAAVATGGGARQLVDASLSIVPRSTP...................
BjTCP15a  .MDPDPDHNPNHHRPNFPLQLLDSSSSSSTSLAIVPSASSS...................
BjTCP15d  .....MDHNPNHHRPNFPLQLLDSS....TSFATIPSASSS...................
BjTCP15c  ...MDPDHNQNHHRPNFPLQLLDSSSSSSTSLAIIPSASSS...................
BjTCP15e  ...MDPDHNQNHHRPNFPLQLLDSSSSSSTSLAIIPSASSS...................
BjTCP15b  ....MDPDPDHHQRPNFPLQLLDSSSSSSTSLAIIPSASSS...................
BjTCP15f  ....MDPDPDHHHRPNFPLQLLDSSSSSSTSLAIIPSASSS...................
AtTCP15   ....MDPDPDHNHRPNFPLQLLDSSTSSSSTSLAIISTTS....................
BjTCP14a  ............................................................
BjTCP14b  GDDHNHHLHHHHHRPTFPFQLLGKRDPDDNNQQQQQPSPSSSSSLFSLHQHQ........
AtTCP14   GGDDHHRHLHHHHRPTFPFQLLGKHDPDDNHQQQPSPSSSSSLFSLHQHQQL........
BjTCP20a  .............MDPKNPNRHQVPNFLNPPPRNQSLGDASKDDK...............
BjTCP20c  .............MDPKNPNRHQVPNFLNPPPRNQSIGDASKD.................
AtTCP20   .............MDPKNLNRHQVPNFLNPPPPPRNQGLVDDDAASAVVSDE........
BjTCP20b  .............MDPKNPNPHQVPNFLIPPPQPRDASDDNKDDN....HHH........
BjTCP6    ........................PNLLNPSE............................
AtTCP6    .............MVMEPKKNQNLPSFLNPSRQNQDNDK.....................
BjTCP1a   ........HQSTASPGQMVSAVPESLIDYMSFNSNSAVNQK...................
BjTCP1e   ........HQSTASPGQMVSAVPESMIDYMSFNSNTAVNQK...................
BjTCP1b   ........HQPTPSPGQMVSAVPESLMNYMSFCSNRAVNQQ...................
BjTCP1c   ........HQPTSSPVQMVSAVPESLINYMSFSSNRAVNQQ...................
BjTCP1d   ........HQSTAYPGQMVSAVPESLINYMSFSSNSAVNQQ...................
AtTCP1    ........HQLKASPGHMVSAVPESLIDYMAFKSNNVVNQQ...................
BjTCP12a  ........HHHYPFPSS...SDFLD...ESVLISQFLLQQQ....ADVDSP.........
BjTCP12b  ........HHHYPLPSFPFSSDFLD...ESVLISQFLLQQQ....DDDNSP.........
AtTCP12   ........HHHYSSPSFPFSSDFLESFDESFLINQFLLQQQDVAANVVESP.........
BjTCP18a  PLD.....HHYQFLQAPSSFSQFEFVPDFALVASFLPQNNGHNDNQTITTNDHNNNHHHH
BjTCP18d  LLDHHHHYHHHQFLQTPSCFSHFESVPDFALLASFLPQNNGHNDNQTITN.....NSHHH
BjTCP18b  HLD.....HHHQFLQAASPFSQFEFVQDFSLLPSFLPQNIAHNDNQTISIN......DHH
BjTCP18c  HLD.....HHHQFLQAPSPFSQFEFVQDFALLPSFLPQNIGHNDNQPITTN......DHH
AtTCP18   HLD......HHHQFQQPSPFSHFEFAPDCALLTSFHPENNGHDDNQTIPND......NHH
BjTCP17a  ....................MGIKQEGDNQYQATSLSSLRQ...................
BjTCP17b  ...................SMGIKQEGDNLYQTTSLSSLRQ...................
AtTCP17   ....................MGIKKE.DQKSSLSLLTQRWN...................
BjTCP5c   ...................MLEQPNQPSFVSSSRQWTSAFR...................
BjTCP5a   ...................HVPAAESASPAPASRQWASAFR...................
BjTCP5b   ...................NMLQQQQNQPVSSSRQWTSAFR...................
AtTCP5    ...................HMLQQQQPSSVSSSRQWTSAFR...................
BjTCP13a  ...................EAVATTKRPATISSSSSWMKSK...................
BjTCP13c  ...................VAVATTKRPASTSSSSSWMKSK...................
BjTCP13b  ..........VVVAVATSGKPVIKKPPTSISSSSSSWMKSK...................
AtTCP13   ..........ETEVRATSGKTVIKKQPTSISSSSSSWMKSK...................
BjTCP24a  ..........DIDEQQQ.TSRKLQRISSDNDKNGMRDWNNP...................
BjTCP24b  ..........DIDAQQQQTSRKLQRISSDNDRNEMRDWNNP...................
BjTCP24c  ..........QQQQQQQQTSRKLQRLSSSDNKTGMRDWSDP...................
BjTCP24d  ..........ETQQEQQQRSRKLQRLSS..DKTEMRDWSNP...................
AtTCP24   ..........ELQKHQEQQSRKLQRFSE.DNTGLMRNWNNP...................
BjTCP2a   ............................................................
BjTCP2d   ............................................................
BjTCP2b   ............................................................
BjTCP2c   ............................................................
AtTCP2    ............MIGDLMKNNNNGDVVDNEVNNRLSRWHHN...................
BjTCP3    ............................................................
AtTCP3    ..........DHFLDSPSPPLLEMRHHQSATENGGGCGEIV...................
AtTCP4    ..........SDDQFHHPPPPSSMRHRSTSDAADGGCGEIV...................
AtTCP16   ............................................................
AtTCP10   ....................MGLKGYSVGEGGGEIVEVQGG...................
```

```
                                          40        50
BjTCP19     .............................QEVGFLQPVTAPKR..........PPSKDRH
AtTCP19     ..............................YLQPVKTKPAPKR..........PTSKDRH
BjTCP9a     .............................LEVPSISMSLAPPSSSIGPPLKR.ASTKDRH
BjTCP9c     .............................LEVPSISMALAPPSSSIGPPLKKRASTKDRH
BjTCP9b     .............................LQVPSTSMSLAPPS....RPPLKRASTKDRH
BjTCP9d     .............................PSLSVSMSLAPPSSSSTMRPPLKRASTKDRH
AtTCP9      ..............................VMPSFSMSLAPPSSTG..PPLKRASTKDRH
AtTCP11     .............................FGVSKSSSSGGGCIS.........ARTKDRH
BjTCP21b    .............................MTNGVIVEHTSATNNKGPLNAVKKPPTKDRH
BjTCP21d    .............................MTNGVTVEQTSAIIK.APLNAVKKPPTKDRH
BjTCP21a    .............................VPNGVIVEHLS.AVNKGTLNAVKKPPAKDRH
BjTCP21e    .............................VPNGVIVEQTSSAVNKGTLNAVKKPPAKDRH
BjTCP21c    .............................VTN.GAIVEHTSAANKGTLNAVKKPPAKDRH
BjTCP21f    .............................VTTNGAIVEHS....KGTLNAVKKPPAKDRH
AtTCP21     .............................VSNGIIVEQTS...NKGPLNAVKKPPSKDRH
BjTCP7a     .............................QRQQQQQQQQNLKQSSDGALVVKKPPAKDRH
BjTCP7e     .............................QRQQQQQQQQ.KQSSTDGALVVKKPPAKDRH
BjTCP7b     ........................................MSNNNDGALVVKKPPAKDRH
BjTCP7d     ........................................MSNSNDGALAVKKPPAKDRH
BjTCP7c     ............................................................
AtTCP7      .............................NNDGLMISSNGALIEQQPSVVVKKPPAKDRH
BjTCP22a    .............AT.................SSSTSTSSGTALAVVKPAAKK.PSKDRH
BjTCP22c    ................................SSSTSTSSGTALAVVKPAAVKKPSKDRH
AtTCP22     .............QHGN..........ISTSATSSTSTSSGSALAVVKSAVKK.PTKDRH
BjTCP22b    ................................GSVP....STALAVVKSAVKK.PSKDRH
BjTCP22d    ................................GSVPSTSSNTALAVVKPAVKK.PSKDRH
AtTCP23     ................................LTTTAPNSTTTTVTAAKTPAKRPSKDRH
BjTCP8a     ..............................PEDPTTSSP.ATAAATTSGVVTKRSTKDRH
BjTCP8b     ..............................PEDPTTSSTSAAAAATTSGVVTKRSTKDRH
AtTCP8      ..............................PEDSTLATTSSTATATTT....KRSTKDRH
BjTCP15a    ...................................EPNSEPNALS.KTPPPKRTSTKDRH
BjTCP15d    ...................................EPNSDPNALS.KKPPPKRTSTKDRH
BjTCP15c    ...................................EPNSDPNALS.KKPPPKRTSTKDRH
BjTCP15e    ...................................EPNSDPNALVSKKPPPKRTSTKDRH
BjTCP15b    ...................................EPNSDPNVLS.KKPPPKRTSTKDRH
BjTCP15f    ...................................EPNADPNALS.KKPPPKRTSTKDRH
AtTCP15     ...................................EPNSEP.....KKPPPKRTSTKDRH
BjTCP14a    ............................................................
BjTCP14b    .............QLS..QTQTQPQQKPQPQKDLQVTQEETAVAVAKKPPLKRASTKDRH
AtTCP14     .............SQSQPQSQSQKSQPQTTQKELLQTQEESAVVAAKKPPLKRASTKDRH
BjTCP20a    .............HHSKPAEVKDFQIVVAS.....DKEPNKKP.QQNQLGPKRSSNKDRH
BjTCP20c    ..............HSKPAEVKDFQIVVAS.....DSSSKKQQNQQNQLGPKRSSNKDRH
AtTCP20     .............NRKPTTEIKDFQIVVSAS....DKEPNKKSQNQNQLGPKRSSNKDRH
BjTCP20b    .............LHNKPAEVKDFQIVVAS..............................
BjTCP6      ................TETEVKKVLNNTVEGKRKGKQGRE..........IQTKSRKDRH
AtTCP6      ..............KRKQTEVKGFDIVVGEKRKKKENEEEDQEIQILYEKEKKKPNKDRH
BjTCP1a     ....................................GYEIPEVSREIKKAVKKDRHSKVH
BjTCP1e     ....................................GFEIPEVPREIKKTVKKDGHSKIH
BjTCP1b     ....................................GFKIHEVSREIKKAVKKDRHSKIQ
BjTCP1c     ....................................GFEIPEASREIKKAVKKDRHSKIQ
BjTCP1d     ....................................GFQIPEVSRELKKAVKKDRHSKIH
AtTCP1      ....................................GFEFPEVSKEIKKVVKKDRHSKIQ
BjTCP12a    ...................RKLRRELEQEKKNEEWVDGTTSEKVSPRRTGKKRDRHSKIC
BjTCP12b    ...................RKLCKELEKEKKKEEWVDGTTSEKVSPRRTGKKRDRHSKIC
AtTCP12     ...................WKFCKKLELKKKNEKCVDGSTSQEVQWRRTVKKRDRHSKIC
BjTCP18a    HPSLLPLNNPIGES....LVEPLETIATHIEDSQRISTSQDPKMNKVKKPSRTDRHSKIK
BjTCP18d    LPSLLPLNSPIGESQLIELIEPSETIITHIEDSQRISTFQDRKVKKVKKPSRTDRHSKIK
BjTCP18b    HPSLLPLNNTIDESQ...LIEPSETITTHNEDSQRISTTQDPKMKNAKKPSRTDRHSKIK
BjTCP18c    HPSLLPLNNTIGESQ...LVEPSENITTHNEDSQRISISQDPKMKKAKKPSRTDRHSKIK
AtTCP18     PSLHFPLNNTIVEQP.....TEPSETINLIEDSQRISTSQDPKMKKAKKPSRTDRHSKIK
BjTCP17a    .......................................NPRIVRASRIFGGKDRHSKVC
BjTCP17b    .......................................NPRIVRASRIFGGKDRHSKVC
AtTCP17     .......................................NPRIVRVSRAFGGKDRHSKVC
BjTCP5c     .......................................NPRIVRVSKAFGGKDRHSKVC
BjTCP5a     .......................................NPRIVRVSRTFGGKDRHSKVC
BjTCP5b     .......................................NPRIVRVSRTFGGKDRHSKVC
AtTCP5      .......................................NPRIVRVSRTFGGKDRHSKVC
BjTCP13a    .......................................DPRIVRVSRAFGGKDRHSKVC
BjTCP13c    .......................................DPRIVRVSRAFGGKDRHSKVC
BjTCP13b    .......................................DPRIVRVSRAFGGKDRHSKVC
AtTCP13     .......................................DPRIVRVSRAFGGKDRHSKVC
BjTCP24a    .......................................SSRIIRVSRASGGKDRHSKVL
BjTCP24b    .......................................SSRIIRVSRASGGKDRHSKVL
BjTCP24c    .......................................SSRIIRVSRASGGKDRHSKVL
BjTCP24d    .......................................SSRIIRVSRATGGKDRHSKVL
AtTCP24     .......................................SSRIIRVSRASGGKDRHSKVL
BjTCP2a     ............................................................
BjTCP2d     ............................................................
BjTCP2b     ............................................................
BjTCP2c     ............................................................
AtTCP2      .......................................SSRIIRVSRASGGKDRHSKVL
BjTCP3      ............................................................
AtTCP3      .......................................EVQGGHIVRSTGRKDRHSKVC
AtTCP4      .......................................EVQGGHIVRSTGRKDRHSKVC
AtTCP16     .......................................MDSKNGINNSQKARRTPKDRH
AtTCP10     ............................................HIIRATGRKDRHSKVF
```

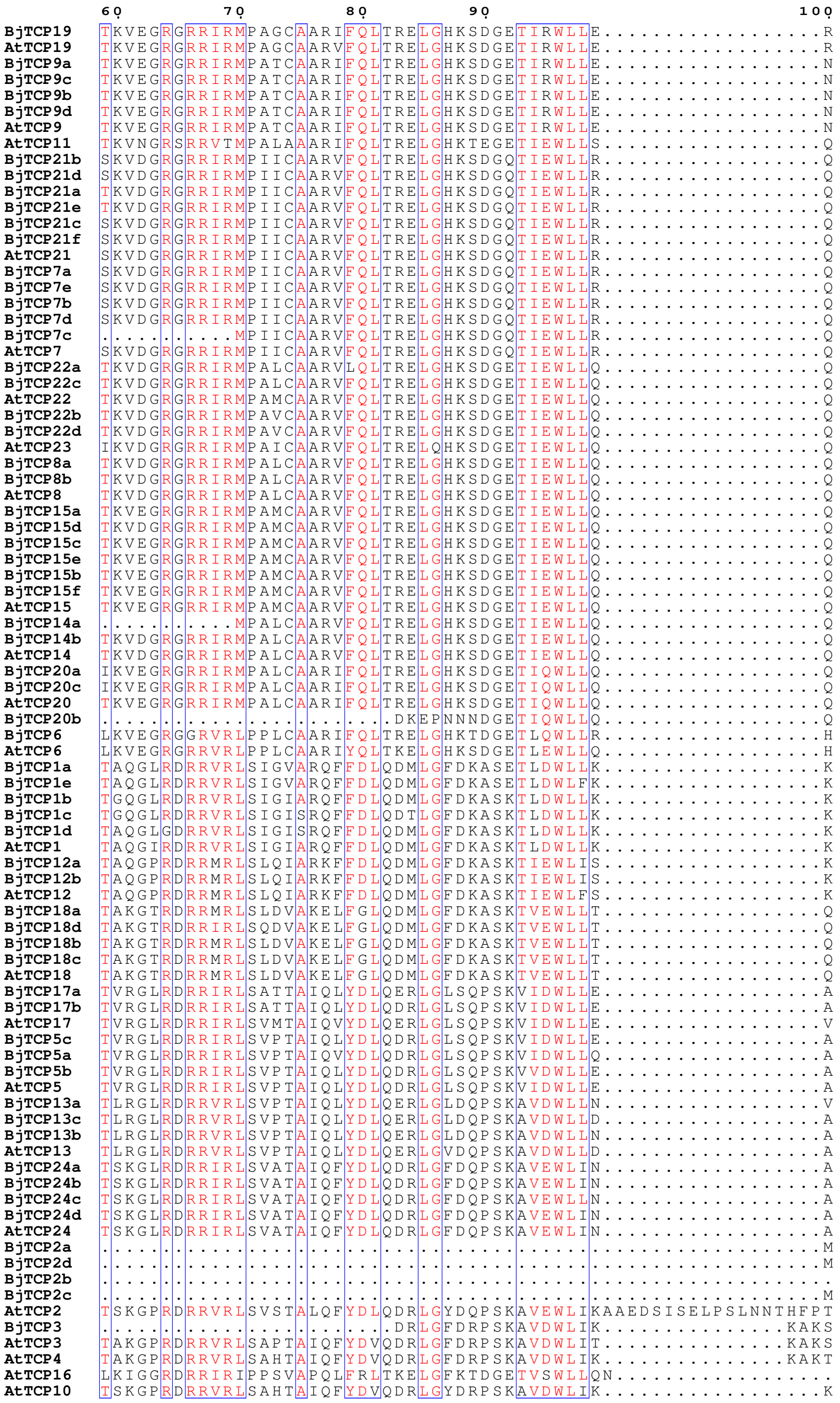
60 70 80 90 100
BjTCP19 TKVEGRGRRIRMPAGCAARIFQLTRELGHKSDGETIRWLLE.................R
AtTCP19 TKVEGRGRRIRMPAGCAARVFQLTRELGHKSDGETIRWLLE.................R
BjTCP9a TKVEGRGRRIRMPATCAARIFQLTRELGHKSDGETIRWLLE.................N
BjTCP9c TKVEGRGRRIRMPATCAARIFQLTRELGHKSDGETIRWLLE.................N
BjTCP9b TKVEGRGRRIRMPATCAARIFQLTRELGHKSDGETIRWLLE.................N
BjTCP9d TKVEGRGRRIRMPATCAARIFQLTRELGHKSDGETIRWLLE.................N
AtTCP9 TKVEGRGRRIRMPATCAARIFQLTRELGHKSDGETIRWLLE.................N
AtTCP11 TKVNGRSRRVTMPALAAARIFQLTRELGHKTEGETIEWLLS.................Q
BjTCP21b SKVDGRGRRIRMPIICAARVFQLTRELGHKSDGQTIEWLLR.................Q
BjTCP21d SKVDGRGRRIRMPIICAARVFQLTRELGHKSDGQTIEWLLR.................Q
BjTCP21a TKVDGRGRRIRMPIICAARVFQLTRELGHKSDGQTIEWLLR.................Q
BjTCP21e TKVDGRGRRIRMPIICAARVFQLTRELGHKSDGQTIEWLLR.................Q
BjTCP21c SKVDGRGRRIRMPIICAARVFQLTRELGHKSDGQTIEWLLR.................Q
BjTCP21f SKVDGRGRRIRMPIICAARVFQLTRELGHKSDGQTIEWLLR.................Q
AtTCP21 SKVDGRGRRIRMPIICAARVFQLTRELGHKSDGQTIEWLLR.................Q
BjTCP7a SKVDGRGRRIRMPIICAARVFQLTRELGHKSDGQTIEWLLR.................Q
BjTCP7e SKVDGRGRRIRMPIICAARVFQLTRELGHKSDGQTIEWLLR.................Q
BjTCP7b SKVDGRGRRIRMPIICAARVFQLTRELGHKSDGQTIEWLLR.................Q
BjTCP7d SKVDGRGRRIRMPIICAARVFQLTRELGHKSDGQTIEWLLR.................Q
BjTCP7c ...........MPIICAARVFQLTRELGHKSDGQTIEWLLR.................Q
AtTCP7 SKVDGRGRRIRMPIICAARVFQLTRELGHKSDGQTIEWLLR.................Q
BjTCP22a TKVDGRGRRIRMPALCAARVLQLTRELGHKSDGETIEWLLQ.................Q
BjTCP22c TKVDGRGRRIRMPALCAARVFQLTRELGHKSDGETIEWLLQ.................Q
AtTCP22 TKVDGRGRRIRMPAMCAARVFQLTRELGHKSDGETIEWLLQ.................Q
BjTCP22b TKVDGRGRRIRMPAVCAARVFQLTRELGHKSDGETIEWLLQ.................Q
BjTCP22d TKVDGRGRRIRMPAVCAARVFQLTRELGHKSDGETIEWLLQ.................Q
AtTCP23 IKVDGRGRRIRMPAICAARVFQLTRELQHKSDGETIEWLLQ.................Q
BjTCP8a TKVDGRGRRIRMPALCAARVFQLTRELGHKSDGETIEWLLQ.................Q
BjTCP8b TKVDGRGRRIRMPALCAARVFQLTRELGHKSDGETIEWLLQ.................Q
AtTCP8 TKVDGRGRRIRMPALCAARVFQLTRELGHKSDGETIEWLLQ.................Q
BjTCP15a TKVEGRGRRIRMPAMCAARVFQLTRELGHKSDGETIEWLLQ.................Q
BjTCP15d TKVDGRGRRIRMPAMCAARVFQLTRELGHKSDGETIEWLLQ.................Q
BjTCP15c TKVEGRGRRIRMPAMCAARVFQLTRELGHKSDGETIEWLLQ.................Q
BjTCP15e TKVEGRGRRIRMPAMCAARVFQLTRELGHKSDGETIEWLLQ.................Q
BjTCP15b TKVEGRGRRIRMPAMCAARVFQLTRELGHKSDGETIEWLLQ.................Q
BjTCP15f TKVEGRGRRIRMPAMCAARVFQLTRELGHKSDGETIEWLLQ.................Q
AtTCP15 TKVEGRGRRIRMPAMCAARVFQLTRELGHKSDGETIEWLLQ.................Q
BjTCP14a ............MPALCAARVFQLTRELGHKSDGETIEWLLQ.................Q
BjTCP14b TKVDGRGRRIRMPALCAARVFQLTRELGHKSDGETIEWLLQ.................Q
AtTCP14 TKVDGRGRRIRMPALCAARVFQLTRELGHKSDGETIEWLLQ.................Q
BjTCP20a IKVEGRGRRIRMPALCAARIFQLTRELGHKSDGETIQWLLQ.................Q
BjTCP20c IKVEGRGRRIRMPALCAARIFQLTRELGHKSDGETIQWLLQ.................Q
AtTCP20 TKVEGRGRRIRMPALCAARIFQLTRELGHKSDGETIQWLLQ.................Q
BjTCP20b ........................DKEPNNNDGETIQWLLQ.................Q
BjTCP6 LKVEGRGGRVRLPPLCAARIFQLTRELGHKTDGETLQWLLR.................H
AtTCP6 LKVEGRGRRVRLPPLCAARIYQLTKELGHKSDGETLEWLLQ.................H
BjTCP1a TAQGLRDRRVRLSIGVARQFFDLQDMLGFDKASETLDWLLK.................K
BjTCP1e TAQGLRDRRVRLSIGVARQFFDLQDMLGFDKASETLDWLFK.................K
BjTCP1b TGQGLRDRRVRLSIGIARQFFDLQDMLGFDKASKTLDWLLK.................K
BjTCP1c TGQGLRDRRVRLSIGISRQFFDLQDTLGFDKASKTLDWLLK.................K
BjTCP1d TAQGLGDRRVRLSIGISRQFFDLQDMLGFDKASKTLDWLLK.................K
AtTCP1 TAQGIRDRRVRLSIGIARQFFDLQDMLGFDKASKTLDWLLK.................K
BjTCP12a TAQGPRDRRMRLSLQIARKFFDLQDMLGFDKASKTIEWLIS.................K
BjTCP12b TAQGPRDRRMRLSLQIARKFFDLQDMLGFDKASKTIEWLIS.................K
AtTCP12 TAQGPRDRRMRLSLQIARKFFDLQDMLGFDKASKTIEWLFS.................K
BjTCP18a TAKGTRDRRMRLSLDVAKELFGLQDMLGFDKASKTVEWLLT.................Q
BjTCP18d TAKGTRDRRIRLSQDVAKELFGLQDMLGFDKASKTVEWLLT.................Q
BjTCP18b TAKGTRDRRMRLSLDVAKELFGLQDMLGFDKASKTVEWLLT.................Q
BjTCP18c TAKGTRDRRMRLSLDVAKELFGLQDMLGFDKASKTVEWLLT.................Q
AtTCP18 TAKGTRDRRMRLSLDVAKELFGLQDMLGFDKASKTVEWLLT.................Q
BjTCP17a TVRGLRDRRIRLSATTAIQLYDLQERLGLSQPSKVIDWLLE.................A
BjTCP17b TVRGLRDRRIRLSATTAIQLYDLQDRLGLSQPSKVIDWLLE.................A
AtTCP17 TVRGLRDRRIRLSVMTAIQVYDLQERLGLSQPSKVIDWLLE.................V
BjTCP5c TVRGLRDRRIRLSVPTAIQLYDLQDRLGLSQPSKVIDWLLE.................A
BjTCP5a TVRGLRDRRIRLSVPTAIQVYDLQDRLGLSQPSKVIDWLLQ.................A
BjTCP5b TVRGLRDRRIRLSVPTAIQLYDLQDRLGLSQPSKVVDWLLE.................A
AtTCP5 TVRGLRDRRIRLSVPTAIQLYDLQDRLGLSQPSKVIDWLLE.................A
BjTCP13a TLRGLRDRRVRLSVPTAIQLYDLQERLGLDQPSKAVDWLLN.................V
BjTCP13c TLRGLRDRRVRLSVPTAIQLYDLQERLGLDQPSKAVDWLLD.................A
BjTCP13b TLRGLRDRRVRLSVPTAIQLYDLQERLGLDQPSKAVDWLLN.................A
AtTCP13 TLRGLRDRRVRLSVPTAIQLYDLQERLGVDQPSKAVDWLLD.................A
BjTCP24a TSKGLRDRRIRLSVATAIQFYDLQDRLGFDQPSKAVEWLIN.................A
BjTCP24b TSKGLRDRRIRLSVATAIQFYDLQDRLGFDQPSKAVEWLIN.................A
BjTCP24c TSKGLRDRRIRLSVATAIQFYDLQDRLGFDQPSKAVEWLLN.................A
BjTCP24d TSKGLRDRRIRLSVATAIQFYDLQDRLGFDQPSKAVEWLIN.................A
AtTCP24 TSKGLRDRRIRLSVATAIQFYDLQDRLGFDQPSKAVEWLIN.................A
BjTCP2a ....M
BjTCP2d ....M
BjTCP2b ....
BjTCP2c ....M
AtTCP2 TSKGPRDRRVRLSVSTALQFYDLQDRLGYDQPSKAVEWLIKAAEDSISELPSLNNTHFPT
BjTCP3 ......................DRLGFDRPSKAVDWLIK...............KAKS
AtTCP3 TAKGPRDRRVRLSAPTAIQFYDVQDRLGFDRPSKAVDWLIT...............KAKS
AtTCP4 TAKGPRDRRVRLSAHTAIQFYDVQDRLGFDRPSKAVDWLIK...............KAKT
AtTCP16 LKIGGRDRRIRIPPSVAPQLFRLTKELGFKTDGETVSWLLQN..................
AtTCP10 TSKGPRDRRVRLSAHTAIQFYDVQDRLGYDRPSKAVDWLIK.................K

```
                  110       120       130       140
BjTCP19    SEPAIIEATGTGTIPAIAVSVNGALKIPTDSTPQFESDGGSG..................
AtTCP19    AEPAIIEATGTGTVPAIAVSVNGTLKIPTSSPVLNDGGRDGDGD................
BjTCP9a    AEPAIIAATGTGTVPAIAMSVNGTLKIPTTTNADADVAENPAK.................
BjTCP9c    AEPSIIAATGTGTVPAIAMSVNGTLKIPTTTNADADLSENPAK.................
BjTCP9b    AEPAIIAATGTGTVPAIAMSVNGTLKIPTTTNADSDVGDNSAK.................
BjTCP9d    AEPAIIAATGTGTVPAIAMSVNGTLKIPTTTNADSDAGDNPAK.................
AtTCP9     AEPAIIAATGTGTVPAIAMSVNGTLKIPTTTNADSDMGENLMK.................
AtTCP11    AEPSIIAATGYGTK..............................................
BjTCP21b   AEPSIIAATGTGTTPASFSTASLSASSSPFALG...KRVLRGEE................
BjTCP21d   AEPSIIAATGTGTTPASFSTASLSSSS.PFTLG...KRVVRAEE................
BjTCP21a   AEPSIIAATGTGTTPASFSTASLSTSS.SFTLG...KRGVRAGE................
BjTCP21e   AEPSIVAATGTGTTPASFSTASLSTSS.SFTLG...KRVVRAGE................
BjTCP21c   AEPSIIAATGTGTTPASFSTASLSTSS.PFTLG...KRVVRAEE................
BjTCP21f   AEPSIIAATGTGTTPASFSSTASSSSS.PFALG...KRVVRAEE................
AtTCP21    AEPSIIAATGTGTTPASFSTASLSTSS.PFTLG...KRVVRAEE................
BjTCP7a    AEPSIIAATGTGTIPASFSTASVSVRGSSTTISTSTNCTLSSSL................
BjTCP7e    AEPSIIAATGTGTTPASFSTASVSVRGNSTTISTSANCTLSSSL................
BjTCP7b    AEPSIIAATGTGTTPASFSTVSASLRGNSTTT...TNCSLSSSL................
BjTCP7d    AEPSIIAATGTGTTPASFSTASASLRGSSTTT...TNCALSPSL................
BjTCP7c    AEPSIIAATGTGTTPASFSTTSTCTIS.............SSSL................
AtTCP7     AEPSIIAATGTGTTPASFSTASVSIRGATNSTS.......LDHK................
BjTCP22a   AEPAIIASTGTGTIPANFSTLNATLRG.GGG.STIFAQSSKSSP................
BjTCP22c   AEPAIIASTGTGTIPANFSTLNATLRG.GGGGSTIFAQSSKSSP................
AtTCP22    AEPAIIASTGTGTIPANFSTLNASLRS.GGG.STLFSQASKSSSSPLS............
BjTCP22b   AEPAIIATTGTGTIPANFSTLNASLRGGGGGGSTVFAQSSKSSSLPMS............
BjTCP22d   AEPAIIATTGTGTIPANFSTLNASLRGGGGGG.TVFAQSSKS..................
AtTCP23    AEPAIIAATGTGTIPANISTLNISLRSSGSTLSAPLSKSFHMGR................
BjTCP8a    AEPAIVAATGTGTIPANFSSLSVSLRSSGSTLSAPPSKSVPLYG................
BjTCP8b    AEPAIVAATGTGTIPANFSSLSVSLRSTGSTLSAPPSKSVPLYG................
AtTCP8     AEPAIVAATGTGTIPANFSTLSVSLRSSGSTLSAPPSKSVPLYG................
BjTCP15a   AEPAVIAATGTGTIPANFASLNISLRSSRSSLSAAHLRT.....................
BjTCP15d   AEPAVIAATGTGTIPANFASLNISLRSSRSSLSAAHLRS.....................
BjTCP15c   AEPAVIAATGTGTIPANFTSLNISLRSSRSSLSAAHLRT.....................
BjTCP15e   AEPAVIAATGTGTIPANFTSLNISLRSSRSSLSAAHLRT.....................
BjTCP15b   AEPAVIAATGTGTIPANFTSLNISLRSSRSSLSAAHLRT.....................
BjTCP15f   AEPAVIAATGTGTIPANFTSLNISLRSSRSSLSAAHLRT.....................
AtTCP15    AEPAVIAATGTGTIPANFTSLNISLRSSRSSLSAAHLRT.....................
BjTCP14a   AEPSVIAATGTGTIPANFTSLNISLRSSGSSMSLPSHYRNAASTFSPNNIFSPSLLQQQQ
BjTCP14b   AEPSVIAATGTGTIPANFTSLNISLRSSGSSMSLPSHYRNAASTFSPNNIFSQSLLQQQQ
AtTCP14    AEPSVIAATGTGTIPANFTSLNISLRSSGSSMSLPSHFRSAASTFSPNNIFSPAMLQQQQ
BjTCP20a   AEPSIIAATGSGTIPASALASAAAAVSSHHLQGGGSLTAGLMISHELDGG..........
BjTCP20c   AEPSIIAATGSGTIPASALASAAAAVSNHHHLQGGSLTAGLMISHELDGGGG........
AtTCP20    AEPSIIAATGSGTIPASALASSAATSN...HHQGGSLTAGLMISHDLDGGSS........
BjTCP20b   AEPSLIAATGSGTVPASALASAASAVVS...NQGGSLTAGLMISHHDLDCG.........
BjTCP6     AEPSIISATDS.......VSQPPPHAVD................................
AtTCP6     AEPSILSATVNGIKPTESVVSQPPLTAD................................
BjTCP1a    SRRGINELVQEKKLNNKDGDYGNNRGGVEDDEED..........................
BjTCP1e    SRRAIKELVQEKKLNNKDDDFGNNRGDVEEDEDD..........................
BjTCP1b    SRRAIKELVQEKKLNNTDEDFGNKGGEVEEEEGD..........................
BjTCP1c    SRRAIKELVKEKKLNNTDKDSGNIGGEVEEEEEEE.........................
BjTCP1d    SRRAIKELVQEKKLSNNDEDFGNNGGNVEKEDDDD.........................
AtTCP1     SRKAIKEVVQAKNLNNDDEDFGNIGGDVEQEEEKEED.......................
BjTCP12a   SKASIKQVKESAAASG......NHEHLQVSDNVND.........................
BjTCP12b   SKASIKQVKESAAGSGGGGGGGNHEHLQVSENAND.........................
AtTCP12    SKTSIKQLKERVAASEGG...GKDEHLQVDEKEKD.........................
BjTCP18a   AKPEIIKITSSLSNPLNHGGFSSCEESQTRPALGSMHTSSDLCKLSSM............
BjTCP18d   AKPEIIKIANSLSNQFNHGPFSSCEESQP...............................
BjTCP18b   AKPEIIKIANSLSNQFKHGGFSSGDDSQTRPALGSTETSSDLCEFASV............
BjTCP18c   AKPEIIKIANSLSNQFNNGGFSSGDDSQTRPALGSMETSSDLCELASV............
AtTCP18    AKPEIIKIATTLS...HHGCFSSGDESHIRPVLGSMDTSSDLCELASM............
BjTCP17a   AQNDVAMLPPLQFPPG....FHPNLTAAAAAG............................
BjTCP17b   AQNDVAMLPPLQFPPG....FHLNLPTATGGAG...........................
AtTCP17    AKNDVDLLPPLQFPPG....FHQLNPNLTGLG............................
BjTCP5c    AKDDVDKLPPLQFPHG....FNQMYPNLIFG.............................
BjTCP5a    AKDDVDNLPPLQFPQG....FNQMYPSLIFG.............................
BjTCP5b    AKDDVDKLPPLQFPHG....FNQMYPNLFFGNSGIG........................
AtTCP5     AKDDVDKLPPLQFPHG....FNQMYPNLIFGNSGFG........................
BjTCP13a   AKEEIDELPPLPVSPETFTLFNHHQSFLNLGQR...........................
BjTCP13c   AKEEIDELPPLPVSPETFTLFNHHQSFLNLGQR...........................
BjTCP13b   AKDEIDELPPLPVSPETFSLFNHHQAFLNLSQP...........................
AtTCP13    AKEEIDELPPLPISPENFSIFNHHQSFLNLGQR...........................
BjTCP24a   AADSISELPPINTSFDQALS...LSKSACSSGT...........................
BjTCP24b   ASDSISELPPINTSFDQALS...LSKSACSSGT...........................
BjTCP24c   ASDSISDLNPIDTNFD.......QTLSLSKSST...........................
BjTCP24d   ASDSISELNPIDTNFD.......QTLSLSKSST...........................
AtTCP24    ASDSITDLPLLNTNFDHLDQNQNQTKSACSSGT...........................
BjTCP2a    NDDENQIQTVTGASP......AAAAKSACSSNS...........................
BjTCP2d    TDDENQNQTVT..........AAAAKSACSSNS...........................
BjTCP2b    .MNDDEIQTQTGVS...AAAGNSLSKSACSSNS...........................
BjTCP2c    NDDDNQIPTLTGASPTAAAAANSLSKSACSSNS...........................
AtTCP2     DDENHQNQTLTTVAAN......SLSKSACSSNS...........................
BjTCP3     AIDGLAELPPWNPADAIRQAAAN....KPKRSKTVISPPGIHGGSGEE............
AtTCP3     AIDDLAQLPPWNPADTLRQHAAAAANAKPRKTKTLISPPPPQPEETEH............
AtTCP4     SIDELAELPPWNPADAIRLAAAN.AKPRRTTAKTQISPSPPPPQQQQQQQQLQFGVGF..
AtTCP16    AEPAIFAATGHGVTTTSNEDIQPNRNFPSYTFNG..........................
AtTCP10    AKTAIDKLELGETTTTTTRQEPVNTKPESPTLVFQRENNDQTQFVAAN............
```

```
                                          150       160
BjTCP19   ...........................KRRRRNCTSEFVDINR.QDSSVTS.........
AtTCP19   ........................LIKKRRKRNCTSDFVDVNDSCHSSVTS.........
BjTCP9a   ...........................KRRKRPSNSEYIDINEPVSVSVSS.........
BjTCP9c   ...........................KRRKRPSNSEYIDINEAASVSS...........
BjTCP9b   ...........................KKRKRPSNSEYIDINEAVTVSVSSGLAPISSGL
BjTCP9d   ...........................KRRKRPSNSEYIDINEAVSVSVSSGLAPISSGL
AtTCP9    ...........................KKRKRPSNSEYIDISDAVSASSGLAPIATTTTI
AtTCP11   .................................LISNWVDVAADDSSSSSS.........
BjTCP21b  ..........................GESVGPGGGGELTVGHAMS...............
BjTCP21d  ..........................GESGGGGGGGHTVGASLMS...............
BjTCP21a  ..........................GEAGAGGGG.LTVGPTMG...AS...........
BjTCP21e  ..........................GVAGAGGGGGLTVGHTMG...AS...........
BjTCP21c  ..........................GESGAGGG.ELSVGHTMG................
BjTCP21f  ..........................EESGGGGGGELSVGHTMGGSVM............
AtTCP21   ..........................GESGGGGGGGLTVGHTMGTSLMG...........
BjTCP7a   ..........................DHK......PFILGKRLREDSGGGKDD.......
BjTCP7e   ..........................DHKPLLGSSPFILGKRLRADEVSGKDESH.....
BjTCP7b   ..........................DQKPMLGSSPFILGKRVRADED............
BjTCP7d   ..........................DHKPLLGSSPFILGKRVRADED............
BjTCP7c   ..........................DHKPLLGSSPFILGKRVREGFGG...........
AtTCP7    ..........................PTSLLGGTSPFILGKRVRADEDSNNSHNHS....
BjTCP22a  FHSTGMSLYEDSNGTNGSSSSAAKLLN.AATAAQNAAVFGFHHQGYQPIMSAERNP..PT
BjTCP22c  FHSTGMSLYEDSNGANGSSSSAVVDPSRKLLNAQNAAVFGFHHQVYPPIT........PT
AtTCP22   FHSTGMSLYEDNNGTNGSS...VDPSRKLLNSAANAAVFGFHHQMYPPIMSTERNP..NT
BjTCP22b  FHGSGMSLYDDSNGANGSSSSVVVDHSRKLLNGG...VFGFHHPPIMSAE..........
BjTCP22d  FHSSGMSLYDDSNGANGSSSSAVVDHSRKLLNGG...VFGFHHPPIMSAE..........
AtTCP23   ...............................AAQNAAVFGFQQQLYHPHHITTDSSSSSL
BjTCP8a   ............ALGLTHHQYEEQ...GGAFGAHTPPLLGFHHHLQQHQQQQHHQQA.PA
BjTCP8b   ............ALGLSHHHHQYEEQGGGAFGPHTPPLLGFHHHLQQQQ..QLHQQA.PA
AtTCP8    ............ALGLTHHQYDEQG.GGGVFAAHTSPLLGFHHQLQHHQNQNQNQDP.VE
BjTCP15a  ..................................APSSYYFHSPTM........AHPHQV
BjTCP15d  ..................................NPSSYYFHP..............HQV
BjTCP15c  ..................................TPSSYYFHSPTM........AHHHQV
BjTCP15e  ..................................TPSSYYFHSPTM........AHHHQV
BjTCP15b  ..................................TPSNYYFHSPAMAQHHH..QQQLHQV
BjTCP15f  ..................................TPSSYYFHSPTMSQHHHQQQQQNHHV
AtTCP15   ..................................TPSSYYFHSPHQSMTHH..LQHQHQV
BjTCP14a  Q.RSGGGGFLHPHHHLQGRATTSSLFPGIDNFTPTTSFLNFHNPTKQEGDQDSEELSPDK
BjTCP14b  QQRSGGGGFLHPHHHLQGRATTSSLFPGIDNFTPTTSFLNFHNPTKQEGDQDSEELSSDK
AtTCP14   Q.QQRGGGVGFHHPHLQGRAPTSSLFPGIDNFTPTTSFLNFHNPTKQEGDQDSEELNSEK
BjTCP20a  ................................SSSGRPNWGVGGGDGGS...........
BjTCP20c  ................................SSSGRPNWGVGGGEGGAS..........
AtTCP20   ................................SSGRPLNWGIGGGEGVS...........
BjTCP20b  ..................................GGRPSWVEGGGEASR...........
BjTCP6    ......................................LNLDGGSRSQ............
AtTCP6    ......................................LMICHSVEEASR..........
BjTCP1a   .......................GDKSFVYGWSPD.SCEEVVYEVKKTEKS.......NV
BjTCP1e   .......................GDKSFVYGLSPD.SCEEVVCEVKKTAVKS......NI
BjTCP1b   ......................DGDKSFLYGSSPDSWEEEVACEVKKAQKRNKKIDLSNI
BjTCP1c   .....................EDGDESFVYGSSPDSCKEEVVCEVKKAEKRKKNIELSNI
BjTCP1d   .....................DVGDKSFVYGSSSDCFKEEVVCEVKKPEKRNKKIELSNI
AtTCP1    .....................DNGDKSFVYGLSPGYGEEEVVCEATKAGIRKKKSELRNI
BjTCP12a  ........................ETQKVSK..GRTKRVDDSCKKKQSREKAR.......
BjTCP12b  ........................ETLKVSM..RRAKRVEDSCKKKESREKAR.......
AtTCP12   ........................ETLKLRVSKRRTKTMESSFKTKESRERAR.......
BjTCP18a  ...................GTVEDRGSNTNSTETRGNKVDGRSMRGKRKMLQARTPILKK
BjTCP18d  ................................QTRGNKVDGRSVRGKRRMSQPRTPILKK
BjTCP18b  ...................WTVEDRGSN...TSMTENKVDGRTMRGKRKMSQR.TPILKE
BjTCP18c  ...................WTVEDRSSN...TSMTENKVDGRTMRGKRRMSQR.TPILKE
AtTCP18   ...................WTVDDRGSNTNTTETRGNKVDGRSMRGKRKRPEPRTPILKK
BjTCP17a  ..........................ESFPGIFESFELGSCSSRTDT........TQKEG
BjTCP17b  ..........................ESFPGIFENFDLGSCSSRAET........TQRES
AtTCP17   ..........................ESFPGVFDLGRTQREALDLEK........RKWVN
BjTCP5c   ..............ESSSTSAFPGTNYTNLGFLQSWDLGGSSSRTRSRITDTTTTL.RES
BjTCP5a   ..............ESSSTSAASAFPGTNLGFLESWNLGGSSSRTRSRIADTTP...RES
BjTCP5b   ..............ESSSSTS..AFPGSNLGFLESYDLGGSSSRTRSRIQDTTTTTPRET
AtTCP5    ..............ESPSSTTSTTFPGTNLGFLENWDLGGSS.RTRARLTDTTTTQ.RES
BjTCP13a  ......................PGQDPTQLGFKINGCVEESTTTSREENNNERGEKDVSF
BjTCP13c  ......................PGQDPIQLGING...STTTTTSSREENNKERGEKDVTF
BjTCP13b  ......................PGQDPTQLGFKINGCVEESNTTTSREE...........
AtTCP13   ......................PGQDPTQLGFKINGCVQKSTTTSREENDREKGENDVVY
BjTCP24a  .....................SES...SLLSLSRTESRGKARERARERTAKDK...DKDL
BjTCP24b  .....................SES...SLLSLSRTESRGKARERARDRTAKEK...DKDL
BjTCP24c  .....................SES...SLLSLSRTESHRGKRTSKEK............D
BjTCP24d  .....................SES...SLLSLSRTESRGKARERTAK............D
AtTCP24   .....................SES...SLLSLSRTEIRGKARERARERTAKDR...DKDL
BjTCP2a   .....................DTSKNSSGLSLSRSELRDKARERARERTAKETKERDHN.
BjTCP2d   .....................DTSKNSSGLSLSRSELRDKARERARERTAKETKERDH..
BjTCP2b   .....................DTSKNSSGLSLSRSELRDKARERARERTAKETKERDGTN
BjTCP2c   .....................DTSKNSSGLSLSRSELRDKARERARERTAKETKERDGNN
AtTCP2    .....................DTSKNSSGLSLSRSELRDKARERARERTAKETKERDHN.
BjTCP3    ...........TGHHHSN......................FLPASVS..........TPH
AtTCP3    ...........HRIGEEEDNESSFLPASMDSDSIADTIKSFFPVAST..........QQS
AtTCP4    .........NGGGAEHPSNNESSFLPPSMDSDSIADTIKSFFPVIGSSTEAPSNHNLMHN
AtTCP16   ............................................................
AtTCP10   .....................LDPEDAMKTFFPATTTTNGGGGTNINFQNYPHQDDNNMV
```

```
                                 170
BjTCP19    ....................GLAPITASSYGVN..........................L
AtTCP19    ....................GLAPITASNYGVN..........................I
BjTCP9a    .....GLAPASTVAQQTLPQGMIPMWAFPSNAV..........................V
BjTCP9c    .....GLAPTSTVAQQTLQQGMIPMWAIPSNAV..........................V
BjTCP9b    APISSGLAPVTTVAQQTLPQGMIPMWAIPSNAV..........................I
BjTCP9d    APISSGLASVSTVSQQTLPQGMIPMWAIPSNAV..........................I
AtTCP9     QP..PQALASSTVAQQLLPQGMYPMWAIPSNAM..........................I
AtTCP11    ............................................................
BjTCP21b   ...........................................................G
BjTCP21d   ...........................................................S
BjTCP21a   ...........................................................L
BjTCP21e   ...........................................................L
BjTCP21c   ...........................................................A
BjTCP21f   ...........................................................S
AtTCP21    ...........................................................G
BjTCP7a    ........................................................MGSF
BjTCP7e    ......................................................ETMGSF
BjTCP7b    ............................................................
BjTCP7d    ............................................................
BjTCP7c    ............................................................
AtTCP7     ...................................................SVGKDETFT
BjTCP22a   LGKPYREDYFKESE..PSGSSQKPGQFQEQEMGPGPGMGRG..TANVVPQPMWAVAPGTT
BjTCP22c   LGKPYREDYFKESE..PSGSSQKPGQFQEQEMGPGTGVGRG..TANVVPQPMWAVAPGST
AtTCP22    LVKPYREDYFKEPS..SAAEPSESSQKASQFQEQELAQGRG..TANVVPQPMWAVAPGTT
BjTCP22b   .RNPYREDYFNKESEVPSGSSQKPGQFQDQELGPGPGMGRGTPTASVVPQPMWAVAPGST
BjTCP22d   .RNPYREDYFKESEVVPSGSSQKPGQFQDQELGPGMGMG....MGRGVPQPMWAVAPGST
AtTCP23    PKTFREEDLFKDPNFLDQEPGSRSPKPGSEAPDQDPGSTRSRTQNMIPPMWALAPTPAST
BjTCP8a    ETIPGPDGESFSRKRYRSVDSSKEDGEGKQNESKSLKESEPPAAAATGAPMWAVAPTNRS
BjTCP8b    ETD....GEGFSRKRYRSE..KEDDEGKQQSESKSLKESESPAAAATGAPMWAVAPTNRS
AtTCP8     TIP...EGENFSRKRYRSVDLSKENDDRKQNENKSLKESE..TSGPTAAPMWAVAPPSRS
BjTCP15a   RPKHESHSSSSS..QLLDHNQMGNYLVQSTAGS.......................LPTS
BjTCP15d   RPKNETHSSSSS..QLLDHNQMGSYLVQSAAGS.......................LPTS
BjTCP15c   RPKNESHSSSSS..QLLDHSQMGNYLVQSTAGS.......................LPTS
BjTCP15e   RPKNESHSSSSSS.QLLDHSPMGNYLVQSTAGS.......................LPTS
BjTCP15b   RPKNESDSCSSS..QLVDHNQMGNYLVQSTAGS.......................LPTS
BjTCP15f   RPKNESHSCSSS..QLVDHNQMGNYLVQSTAGS.......................LPSS
AtTCP15    RPKNESHSSSSSSSQLLDHNQMGNYLVQSTAGS.......................LPTS
BjTCP14a   KRRLQTTSDLH.....HQHDQIGGYTLQSSNSG.....................STATTA
BjTCP14b   KR...RLQDLH.....HQHDQIGAYTLQSSNSG.....................STATTA
AtTCP14    KRRIQTTSDLHQQQQQHQHDQIGGYTLQSSNSGST..................ATAAAAQ
BjTCP20a   ..........................................................RS
BjTCP20c   ..........................................................RS
AtTCP20    ..........................................................RS
BjTCP20b   ............................................................
BjTCP6     ............................................................
AtTCP6     ..........................................................TQ
BjTCP1a    SSKGLMDKARGKSKQITREMV.YDHPEAVSEITQ..........................
BjTCP1e    SSKGLREKARGKSKQITREMT.YDHPETISEITQ..........................
BjTCP1b    SSKGSRAKARGKAKELT.....YDHPETISEITQ..........................
BjTCP1c    SSKGSRAKARGKAKEMT.....YDHPETISEISQ..........................
BjTCP1d    SSKRSRAKALGKAKEIAREMI.SDHPETASEIIQ..........................
AtTCP1     SSKGLGAKARGKAKERTKEMMAYDNPETASDITQ..........................
BjTCP12a   ....ERARERTMTKMKMRLSGLIDTSKTFADPNQ..........................
BjTCP12b   ....ERARERTMTKMKMRLTGLMDTSKTFSDPNQ..........................
AtTCP12    ....KRARERTMAKMKMRLF...ETSETISDPHQ..........................
BjTCP18a   LSKDERAKARERAKDRTKEKLMKRR...SQVTVL..........................
BjTCP18d   LSKDARAKARERAKDRTKEKMMKRS...AQVNVV..........................
BjTCP18b   MSKDARAKARERAKDRTMEKIMKRRR.PQVNAVE..........................
BjTCP18c   MSKDARAKARERAKDRTMEKMMKRR..PQVNAVE..........................
AtTCP18    LSKEERAKARERAKGRTMEKMMMKMKGRSQLVKV..........................
BjTCP17a   LNLES....HGFDIDHHFFSNSNHRDKLYFPSSR..........................
BjTCP17b   LNLERNSLSHGFDIDHHFYSNSSQSNNKVYLPTL..........................
AtTCP17    LDHVFD......HIDHHNHFSNSIQSNKLYFPTI..........................
BjTCP5c    FDLDKGKWIKHDENSNHHQEHGFDMNHNHFSLTN..........................
BjTCP5a    FDLDKGKWIKQDENSNHDQDLGFNTSHQHFSLTN..........................
BjTCP5b    FDLDKGKWIKHDENTNHHQDHGFDTNHQHFSLSN..........................
AtTCP5     FDLDKGKWIKNDENSNQDHQGFNTNHQQQFPLTN..........................
BjTCP13a   ANNHHIGSYGTYHNMEHHHQQHS..SFQADYHQH..........................
BjTCP13c   ANNHHIGSYGTYN.MEHHHQQHS..SFQADYHQH..........................
BjTCP13b   TNNNHIGSYGTYQYMEQHQQQHTRFQADYPHHQH..........................
AtTCP13    TNNHHVGSYGTYHNLEHHHHHHQHLSLQADYHSH..........................
BjTCP24a   QNAQSS.FTQLLTGGFDEPNRNWIGG..SSSDCF..........................
BjTCP24b   QNADQSSFTQLLTGGFDEPNRNWIGGGSSSSDCF..........................
BjTCP24c   KDLQSS.FTQLLTTGLDEPNRNWTGAG..SSDCF..........................
BjTCP24d   KDLQSS.FTQLLTRGLDEPNRSWTGGG..STDCF..........................
AtTCP24    QNAHSS.FTQLLTGGFDQQPSNRNWTG..GSDCF..........................
BjTCP2a    ...TTTSFTDLLNSGSDPVNINRQWMAPSSSSSQ..........................
BjTCP2d    ....TGSFTDLLNSGSDPVNVNRQWMATSSSPAQ..........................
BjTCP2b    NHHTTTSFTDLLNSGSDPVNTNRQWMAPSSSPAQ..........................
BjTCP2c    NHHTTS.FTDLLNSGSDPVNANRQWMAPSSSPAP..........................
AtTCP2     ....HTSFTDLLNSGSDPVNSNRQWMASAPSSSP..........................
BjTCP3     YHQPPP....SRADAQSQ.DLRLSLHSFQNGPP...........................
AtTCP3     YHHQPP....SRGNTQNQDLLRLSLQSFQNGPP...........................
AtTCP4     YHHQHPPDLLSRTNSQNQ.DLRLSLQSFPDGPPSLLHHQHH................HHT
AtTCP16    ............................................................
AtTCP10    SRTTTPPPNLSQDLGLSLHPFQGNNNTVVVPETN.........................N
```

```
             180       190           200
BjTCP19    MNVNAQSFVPFWQT....GFVTGGPN..................................
AtTCP19    LNVNTQGFVPFWPMGMGTAFVTGGPD..................................
BjTCP9a    PTVGAFFLVQNPSN...QPRILAYPA..................................
BjTCP9c    PTVGAFFLVPHVAVPLSQPQMLAFPA..................................
BjTCP9b    PTVGAFFLVPHVAGPTNQPQMLAFPA..................................
BjTCP9d    PTVGAFFLVPHVAGPTNQPQMLAFPA..................................
AtTCP9     PTVGAFFLIPQIAGPSNQPQLLAFPA..................................
AtTCP11    .....................MTSPQ..................................
BjTCP21b   GGGGGFWAVPARQDFG............................................
BjTCP21d   GSGGSFWAVPARQDFG............................................
BjTCP21a   MSGGGFWAVPARPDFG............................................
BjTCP21e   MSGGGFWAVPARPDLG............................................
BjTCP21c   SGGGGFWAVPARNDFG............................................
BjTCP21f   GGSGGFWAVPARPDLG............................................
AtTCP21    GGSGGFWAVPARPDFG............................................
BjTCP7a    ATPAGFWAVPARPDFG............................................
BjTCP7e    AAPAGFWAVPARPDFG............................................
BjTCP7b    .TPSGFWAVPARQEFG............................................
BjTCP7d    .TPSGFWAVPARPEFG............................................
BjTCP7c    AIPAGFWSVPARPEFG............................................
AtTCP7     TTPAGFWAVPARPDFG............................................
BjTCP22a   NGGSAFWMLPMSGSGGREQMQQQ.....................................
BjTCP22c   NGGSAFWMLPMSGSGGREQMQQQ.....................................
AtTCP22    NGGSAFWMLPMSGSGGREQMQQQ.....................................
BjTCP22b   NGGSAFWMLPMSGSGGREQMQQQQ....................................
BjTCP22d   NGGSAFWMLPVSGSGGREQMQQQQ....................................
AtTCP23    NGGSAFWMLPVGGGGG..PANVQD....................................
BjTCP8a    AGGNTFWMLPVPTTAAGNQPAAMESSSNAS.RAHMWP..FGGGG..AGAGGGGATHFMAG
BjTCP8b    AGGNTFWMLPVPTTTAGNQPAAMESSSNANNRAHMWP..FGAGAGAGGGGGGGATHFMSG
AtTCP8     GAGNTFWMLPVPTTAGNQMESSSNNNTAAGHRAPPMWPFVNSAGGGAGGGGGAATHFMAG
BjTCP15a   QTPTTAPFWSSGDS..............................................
BjTCP15d   QTPTPAPFWSSGDS..............................................
BjTCP15c   QTPATAPFWSSGDN..............................................
BjTCP15e   QTPATAPFWSSGDN..............................................
BjTCP15b   QTPATAPFWSSGDN..............................................
BjTCP15f   QTPATAPFWSSGDNN.............................................
AtTCP15    QSPATAPFWSSGDN..............................................
BjTCP14a   QIPGNFWMVAAAAAAGG....NNNQTGGGSVGSGGGS.......................
BjTCP14b   QIPGNFWMVAAAGGN......NNQTGGGSVGSAGG.........................
AtTCP14    QIPGNFWMVAAAAAAGGGGGNNNQTGGLMTASIGTGG.......................
BjTCP20a   SLPTGLWPNVAGFGAG............................................
BjTCP20c   SLPTGLWPNVAGFGAAG...........................................
AtTCP20    SLPTGLWPNVAGFGSG............................................
BjTCP20b   .....LWPNGAG................................................
BjTCP6     .LANGFWRNETGFGIV............................................
AtTCP6     MEANGLWRNETG................................................
BjTCP1a    ...........................................................T
BjTCP1e    ...........................................................T
BjTCP1b    ...........................................................T
BjTCP1c    ...........................................................T
BjTCP1d    ...........................................................T
AtTCP1     ...........................................................S
BjTCP12a   ...........................................................V
BjTCP12b   ...........................................................E
AtTCP12    ...........................................................E
BjTCP18a   ...........................................................D
BjTCP18d   ...........................................................V
BjTCP18b   ...........................................................E
BjTCP18c   ...........................................................E
AtTCP18    ...........................................................V
BjTCP17a   SCH.........................................................
BjTCP17b   STYNSSSSCHYNLG............................................QL
AtTCP17    TSS..SSSYHYNLG............................................HL
BjTCP5c    PYNNSNTSSYYNLG............................................HL
BjTCP5a    LYNNNNASSYYNLG............................................HL
BjTCP5b    PYNNNNTSSYYNLG............................................HL
AtTCP5     PYNN..TSAYYNLG............................................HL
BjTCP13a   QLHSLVPFPSQFLV............................................CP
BjTCP13c   QLHSLVPFPSQFLV............................................CP
BjTCP13b   QLHSLVPIQSQFLV............................................CP
AtTCP13    QLHSLVPFPSQILV............................................CP
BjTCP24a   NPVQLIPNSSSSS.............LHHYN.......................TNNHRQ
BjTCP24b   NPVQLIPTTSSSSS............LHHFN.......................SNQTSL
BjTCP24c   NPVQLIP................................................ADSLN
BjTCP24d   NPVQLMPNS..................LNHR.......................QEPSLN
AtTCP24    NPVQLQIP................................................NSSS
BjTCP2a    MEYLSSGLILGSGQ.THFPVQTNAHP...............................FSD
BjTCP2d    MEYLTSGLILGSGQ.THFPIQTSAHP...............................FSD
BjTCP2b    MEYFSSGLILGSGQ.THFPIQTNSHPFLAI.......................SDQHHH
BjTCP2c    MEYFSSGLILGSGQ.THFPIQTNSHP............................FDHHHH
AtTCP2     MEYFSSGLILGSGQQTHFPISTNSHPFSSIS.......................DHHHHH
BjTCP3     FSDQTEHALFSGQSN.PLVFDSSASSWDQSQ.......................QN....
AtTCP3     FPNQTEPALFSGQSNNQLAFDSSTASWEQSH.......................QSPEFG
AtTCP4     SASASEPTLFYGQSN.PLGFDTSSWEQQSSE.......................FG....
AtTCP16    ............................................................
AtTCP10    FTTTHFDTFGRISGWN............................................
```

```
                       210         220         230
BjTCP19    ..QMGHMWAIPTVAAPSFLNGGGTRPVPSYVSN...........................
AtTCP19    ..QMGQMWAIPTVATAPFLNVG.ARPVSSYVSN...........................
BjTCP9a    ..ATAALPATYVAAVQQASSMARPPPPPQVVP............................
BjTCP9c    ..SAAASPSTYDAAVQQAPSMSRPPPSPRVVPSNGV........................
BjTCP9b    ..APP...SSYVAAVQQASLMARPPFVSVSTPS...........................
BjTCP9d    ..APPQ..SSYVAAVQQASSMARPPFPRVPDVT...........................
AtTCP9     ..AAAS.PSSYVAAVQQASTMARPPPLQVVPSSGFVS.......................
AtTCP11    ..TQTQTPQSPSCRLDLCQPIGIQYPVNGYSHMP..........................
BjTCP21b   .....QVWSFAAGATPEMLFTQQQQ..............................AATLF
BjTCP21d   .....QVWSFAAGATPEMLFTQQQQ..............................AATLF
BjTCP21a   .....QVWSFAAGAPPEMLFTQQQQ..............................AATLF
BjTCP21e   .....QVWSFSGASPEMLFAQQQQQ..............................PATLF
BjTCP21c   .....QVWSFAAGAPPEMLFTQQQQQ.............................PATLF
BjTCP21f   .....QVWSFAAGAPPEMLFTQQQQQ.............................PATLF
AtTCP21    .....QVWSFATGAPPEMVFAQQQQ..............................PATLF
BjTCP7a    .....QVWSFAS..HQEMFLQQQQQQQ..........................PAAAALF
BjTCP7e    .....QVWSFAS..HQEMFLQQQQQQQQ.......................QPAAAAALF
BjTCP7b    .....QVWSFAAGATQEMLLQQQQQQ............................AAGLFV
BjTCP7d    .....QVWSFAAGATQELFLQQQQQQQ..........................AAALFVH
BjTCP7c    .....QVLSFAAGAPHEMFLQQHQQQE............................AAALF
AtTCP7     .....QVWSFAGAPQEMFLQQQHHHQQP..............................LF
BjTCP22a   ..PGHQMWAFNPGSYPVGTGRVVTAP.............................MGSMM
BjTCP22c   ..PGHQMWAFNPGSYPVGTGRVVTAP.............................MGSMM
AtTCP22    ..PGHQMWAFNPGNYPVGTGRVVTAP.............................MGSMM
BjTCP22b   ..PGHQMWAFNPGSYPVGTGRVVTAAP............................MGSMM
BjTCP22d   ..PGHQMWAFNPGSYPVGTGRVMTAP.............................MGSMM
AtTCP23    ..PSQHMWAFNPGHYPGRIG....SVQ............................LGSML
BjTCP8a    TGFSFPMDQYRGSPLQLGSFLAQPQQ..........................PNQNIGLS
BjTCP8b    TGFSFPMEQYRGSPLQLGSFLAQPQQQQ.......................QPNQNIGLS
AtTCP8     TGFSFPMDQYRGSPLQLGSFLAQPQP...........................TQNLGLS
BjTCP15a   ...TQNLWAFNINPHHSGEVYNTSGGGGGGGGGGGAGGGG.......VHLMNFAAPIALF
BjTCP15d   ...TQNLWAFNINPHHSGDAYNTSGGGGGGGGG..............VHLMNFAAPIALF
BjTCP15c   ...TQNLWAFNINPHHSADVYNTNGGGGGGGGG.....AG.......VHLMNFAAPIALF
BjTCP15e   ...TQNLWAFNINPHHSADVYNTNGGGGGGGGGGGGGGAG.......VHLMNFAAPIALF
BjTCP15b   ...TQNLWAFHHSGDVYNTSSGGGGGGGGAGGG.....GG.......VHLMNFPAPFALF
BjTCP15f   ...TQNLWAFNAN....PHHSGDVYNTSGGVGG.....GG.......VHLMNFPAPIALF
AtTCP15    ...TQNLWAFNINPHHSGVVAGDVYNPNSGGSGGG...SG.......VHLMNFAAPIALF
BjTCP14a   .GGGEPVWTFPSINTAAAALYRSSVS...GGAVS....SG.......LHFMNFAAPMAFL
BjTCP14b   ...GEPVWTFPSINTAAAALYRSSVSGVSGGAVS....SG.......LHFMNFAAPMAFL
AtTCP14    .GGGEPVWTFPSINTAAAALYRSGVSGVPSGAVS....SG.......LHFMNFAAPMAFL
BjTCP20a   ....VQTMSDGGGYRIGFPGFDYPGGAMSFASILGGG..............SNNQMPGLE
BjTCP20c   .VQTTGMMSDGAGYRIGFPGFDYPGGAMSFASILGGN..............NNNQMPGLE
AtTCP20    .VPTTGLMSEGAGYRIGFPGFDFPGVGHMSFASILGG..............NHNQMPGLE
BjTCP20b   .....GVMSEGAGYRIGFPGFDFPGGAMSFASIFGAGGG...........GNGNHMLGLE
BjTCP6     .GANPTGGGFDLNCGIGFLGFDFNG.....SSDMGFE...............NNQMP...
AtTCP6     ....QTIGGFDLNYGIG...FDFNG.....VPEIGFG...............DNQTPGLE
BjTCP1a    EIMDPFKRSIIFNEGDDMTHSFYKEAIQEFDNQE................CILTKTNVNL
BjTCP1e    KIMDPSKRSIISNEGGDMTHSFYKEAIEEFDNQE................CILTKTKVNL
BjTCP1b    ETIDPLKRSIISNEGEHMTHSLYKEATQEFDSQE................CILSKTKVNL
BjTCP1c    DIIDPLKRSIIFNEGELMKHSLYKEATQEFDSQES...............CILSKMKVNL
BjTCP1d    EIVDPFKRSIIFNEGEHMTDSFYKESIQESGNQE.................CTTKMKVNL
AtTCP1     EIMDPFKRSIVFNEGEDMTHLFYKEPIEEFDNQE..................SILTNMTL
BjTCP12a   SRKTKIIGGAQER.ENLEQER................................SIIEKFL
BjTCP12b   SRKTKITGGAQVEGQNLEQERSTTN......LNR................DSVSIIEKFL
AtTCP12    TREIKITNGVQLLEKENKEQEWSNTNDVHMVEYQM...............DSVSIIEKFL
BjTCP18a   AEAHNQHDEIVKNNKSHVNCKSFEATPCQEETEELLCKN...........DGFAVCNEFV
BjTCP18d   EEEVHNHHDEIVLVKNNK..SNVKATPCEDKIQELLCKN...........DHFPVSNEFV
BjTCP18b   VEAHNQHDEQQ.................................................
BjTCP18c   VEAHNQLDEVVKNNNTGVNWNTTFEVTPCGENMEELCKN...........NRFADYNDFV
AtTCP18    EEDAHDHGEIIKNNNRSQVNRSSFEMTHCEDKIEELCKN...........DRFAVCNEFI
BjTCP17a   ............................................................
BjTCP17b   QQSLLDQSGNVTVALSN...NNNLNPPTVET........................MSSLF
AtTCP17    QQSLLDQSGNVTVAFSNNYNNNNLNPPAAET........................MSSLF
BjTCP5c    QHSLEQSGNNVTVAISNVPNNNNNSLNLPPP.......................AMSSLF
BjTCP5a    QHSLDQSGNNVTVAISNVPNNNNNLNLAPPP.......................TMSSLF
BjTCP5b    QHSLDQSGNNVTVAIPNVPSNNNNNNLNMPPPSSAGDGS......QLFFGPPPPAMSSLF
AtTCP5     QQSLDQSGNNVTVAISNVAANNNNNLNLHPPSSSAGDGS......QLFFGPTPPAMSSLF
BjTCP13a   MTTLSTTTTTTQSLFPSSSSAGSWTMETTDPR......................RMVSHF
BjTCP13c   VTTLS...ATTQSLFPSSSSAGSWTMETTDPR......................RMVSHF
BjTCP13b   VTTSS.TTTTIQSLFPSSSSAGSRTMETTDPR......................QMVSHF
AtTCP13    MTTSP.TTTTIQSLFPSSSSAGSGTMETLDPR......................QM....
BjTCP24a   ETSLNHHLSFVPDYNFGISSSDSPAAAAVNG...................GCYTSRGTLQ
BjTCP24b   NHHHHNHFSFVPDYNFGISSSDSPGAAAG......................CYISRGTLQ
BjTCP24c   HHHSQNQYSFVPNYNFGISSSDSPGAAGC.......................YSGRGTLQ
BjTCP24d   HNNSQNQYSFLPNYNVGVSSSDSPGEAGC.......................YSSRGTLQ
AtTCP24    QEPMNHPFSFVPDYNFGISSSSS.AINGG.......................YSSRGTLQ
BjTCP2a    HHHHHQEFSFVPDHLISPAGSNGGGAFNLDFNMSTTSGA...GTAVASAGFSGFNRGTLQ
BjTCP2d    HHHHPQEFSFVPDHLISPAGSNGGGAFNLDFNMSSTT......SAAGTAGFSGFNRGTLQ
BjTCP2b    PHHPHQEFSFVPDHLISPAGANGGGSFNLDFNMSTTS........VASTGFSGFNRGTLQ
BjTCP2c    HHQPHQEFSFVPDHLISPAGSNGGGAFNLDFNMSTTS........VASTGFSGFNRGTLQ
AtTCP2     PHHQHQEFSFVPDHLISPAESNGG.AFNLDFNMSTPSGAGAAVSAASGGGFSGFNRGTLQ
BjTCP3     ..HRLVTWNNGGAADSAAS.GGGGFVFASPTTTTSFQP..........QSQVFSQRGPLQ
AtTCP3     KIQRLVSWNNVGAAESAGS.TGG.FVFASPSSLHPVYS..........QSQLLSQRGPLQ
AtTCP4     RIQRLVAWNSGGGGGATDTGNGGGFLFAPPTPSTTSFQP.....VLGQSQQLYSQRGPLQ
AtTCP16    ...DNISNNVFPCTVVNTGHRQMVFPVS................................
AtTCP10    .HHDLTMTSSSSSEHQQQEQEERSNGGFMVNHHPHHHHHQPSMMTLLNSQQQQVFLGGQQ
```

```
                                240        250        260        270
BjTCP19    .................TSDAEPQMGTSGGGGDEGTTQTLRDFSLETYDKKELQFLGGSG
AtTCP19    .................ASDAEAEMETSGG....GTTQPLRDFSLEIYDKRELQFLGGSG
BjTCP9a    .............NFTGSVMAPSSSSS......IATKNMLRDFSLESYNKKELHQFMTTT
BjTCP9c    .........ISVPNVNGSVMAPSSSSSSGIANVAPTMNMLRDFSLEIYDKQELHQFMTTT
BjTCP9b    ...............VMAPSSSSG..VTNGGSSSSTTNMLRDFSLEIYEKQELHQFMSTT
BjTCP9d    ...............GSAPSSSSSGITNGGSSSSTTTNMLRDFSLEIYEKQELHQFMSTT
AtTCP9     VSDVSGSNLSRATSVMAPSSSSGVTTGSSSSIATTTTHTLRDFSLEIYEKQELHQFMSTT
AtTCP11    ..................................FTAMLLEPMTTTAESEVEIAEEEERR
BjTCP21b   VRQQQQQ.....ASAAVAAMGEASAARVGNYLPGHHLNLLASLSGGSPGSGRREEDDSR.
BjTCP21d   VRQQQQQQQQQQASAAVAAMGEASAARVGNYLPGHHLNLLASLSGGNTGSGRREEDDSR.
BjTCP21a   VRQQQ.....QQQASAAAAMGEASAARVGNYLPGHHLNLLASLSGGNTGSGRREEDNPR.
BjTCP21e   VRHQQ.....QQQQ.AAAAMGEASAARVGNYLPGHHLNLLASLSGGNTGSGRREEDDPR.
BjTCP21c   VRHQQQQ...QQEASAAAAMGEASAARVGNYLPGHHLNLLASLSGGNTGSGRREEEDDSR
BjTCP21f   VRHQQQQ...QQQASVEAAMGEASAARVGNYLPGHHLNLLASLSGGNTGSGRRDEEDNPR
AtTCP21    VRHQQQQ...QASAAAAAAMGEASAARVGNYLPGHHLNLLASLSGGANGSGRREDDHEPR
BjTCP7a    VHQQQQQ.........QAAMGEASAARVGNYLPG.HLNLLASLSGGAPGSGRREDDQR..
BjTCP7e    VHQQQQQ.........QAAMGEASAARVGNYLPG.HLNLLASLSGGAPGSGRREDDQR..
BjTCP7b    HQQQQQQ.........QAAMGEASAARVGNYLPG.HLNLLASLSSGAPGSGRGEDDDQR.
BjTCP7d    QQQQQQQ.........QAAVGEASAARVGNYLPG.HLNLLASLSSGAPGSGRRVDDEQR.
BjTCP7c    VHQQRQQ.........QGAIGEASAARVGNNLPG.HLSLLASLSGGVPASGRREDDTR..
AtTCP7     VHQQQQQ.........QAAMGEASAARVGNYLPG.HLNLLASLSGGSPGSDRREEDPR..
BjTCP22a   LGGQQLG........LGVAEGNMAAAMRGSRGGGG.DGLAMTLDQHQHQHHQQHQEPNQS
BjTCP22c   LGGQQLG........LGVAESNMAAAMRGSRGGG..DGLAMTLDQHQHHHQHQEP.TTQS
AtTCP22    LGGQQLG........LGVAEGNMAAAMRGSRGDG....LAMTLDQHQHQLQHQEP..NQS
BjTCP22b   LGGQQLG........LGVAEGNMAAALRGGRGGD...GLGMTLEQHQHQHHQQHQEPNNQ
BjTCP22d   LGGQQLG........LGVAETNMAAAMRGGSGGD...GLGMTLEQHQHQHHQQHEEPN.Q
AtTCP23    VGGQQLG........LGVAENNNLGLFSGGGGDGGRVGLGMSLEQKPQHQVSDHATRDQN
BjTCP8a    MPDSNLG.........VLAALNAAYPRGGNANAEQVNN....AVEHQEKQQPQQSDQDDD
BjTCP8b    MPDSNLG.........VLAALNAAYSRGGNANAEQVNNNAVEHQEKQQPQQPQQSDQDDD
AtTCP8     MPDSNLG.........MLAALNSAYSRGGNANANAEQAN....NAVEHQEKQQQSDHDDD
BjTCP15a   SG.QPLASG........YGGGGGDHNHYGVLAALNAAYRPVQET.....ASQQNREGDHH
BjTCP15d   SG.QPLASG........YGGGGGEHNHYGVLAALNAAYRPVQET.....ANQQSREGDHH
BjTCP15c   SG.QPLASGYGGGGGGGGGGGGGEHNHYGVLAALNAAYRPVQET.....ANQQNRDGDHH
BjTCP15e   SG.QPYG..........GGGGGGEHNHYGVLGALNAAYRPIQET.....ANQQNREGDHH
BjTCP15b   SG.QPLASG.....YGGGGGGGGEHNHYGVLSALNAAYRPVQET.....ANQQN.HGDHH
BjTCP15f   SG.QPLASG.....YGGGGGSGGEHNHYGVLAALNAAYRPVQET.....ANQQNRDGDHH
AtTCP15    SG.QPLASG.....YGGGGGGGGEHSHYGVLAALNAAYRPVAETGNH.NNNQQNRDGDHH
BjTCP14a   TG.QQLATSSNHEINEDNNNNEGGRSDGG.DHHNTQRHHHQQQQQQQQHHHHNILSGLNQ
BjTCP14b   TG.QQLATSSNHEINEDNNNNEGGRSDGG.DHHDTQRHHHHHQQQQQ..HHHNILSGLNQ
AtTCP14    TGQQQLATTSNHEINEDSNNNEGGRSDGGGDHHNTQRHHHHQQ.....QHHHNILSGLNQ
BjTCP20a   LGLAQEGNVGVLNPQSFAQIYQQQMSQAQAQGRVLHHTLQHNP.THEEHQQESGEKDDSQ
BjTCP20c   LGLAQEGNVSGVLNPQSFALIQQQMGQAQAQGMVLHHTLHHNPSSHEEHQHESGEKDDSQ
AtTCP20    LGLSQEGNVGVLNPQSFTQIYQQMG...QAQAQAQGRVLHHMHHNHEEHQQESGEKDDSQ
BjTCP20b   LGLSQEGNVGVLN.....QQIYQQMAQAQAQGRVLHHTLHHNP.GHEDHQQESCGRDESQ
BjTCP6     ....QVGVL..............NPRIYQQMGQEQAKVLQHHYHHPHEAQQENADKQGSS
AtTCP6     LRLSQVGVL..............NPQVFQQMGKEQFRVLHHHS...HEDQQQSAEENGS.
BjTCP1a    PTDMDQSYNQHYGTFMLKDKGSSSNYNTILPQNLDYGYDQNPFMDQLFCAVTNRNFPRGK
BjTCP1e    PTDMDRSYNQHYATFMLKGQGSSSNNTTILPQNLEYGYDQNPSMDQPFCAVTNRNFHRGK
BjTCP1b    PTNMDQIYNQNYGTFMLKDQGSSSNYNNILPQNLDFYYDQNSFIDQPFCAVTKTSFPRGN
BjTCP1c    PTNMDQSYNQHYGTFMLKDQGSSSNYNNTPPQNLDFYYDQNPFIDQPFCAVTKTSFPRG.
BjTCP1d    LINMDQNYNQQYRESMLKDQGFSSNYNAILPQNLDFDYNPNPFIDQLFCGVTNTNFFSGI
AtTCP1     PTKMGQSYNQNNGILMLVDQSSSSNYNTFLPQNLDYSYDQNPFHDQTLYVVTDKNFPKGK
BjTCP12a   GLTSESSSSTIFGDSEESYTSLGSIRETVSTTGMVIPREHN...TTSTASVDEERNPIST
BjTCP12b   GLTSESSSSTIFGDSEESYTSLGSIRGTVSTAGVVIPREHN...TASTASVDEERNPTST
AtTCP12    GLTSDSSSSSIFGDSEECYTSLSSVRG......MSTPREHN...TTSIATVDEEKSPISS
BjTCP18a   VN...............KFNSSFPMPNHHRSQGTVSSIE.....QQRQFMDLHHFLERPR
BjTCP18d   MDKKDHISD........EFNSSFPMLDHHHSQWAASSIE.....QQRQFVDLHHFLGKPR
BjTCP18b   ............................................QQQQVRDVHHFLYNYH
BjTCP18c   VNKKDHISDESYNMI.SKLNSSFPVLTHHRSQGVATSIEQQQQQQQQQVRDVHHFLEKPR
AtTCP18    MNKKDHISNESYDLVNYKPNSSFPVINHHRSQGAANSIE.....QHQFTDLHYSFGAKPR
BjTCP17a   ............................................................
BjTCP17b   PRYPLFLEGG..........DQLQLFSSHSNSSKQTDHVE....................
AtTCP17    PTRYPSFLGG..........GQLQLFSSTSSQPDHIE.......................
BjTCP5c    PTYPSFLGASQQHHHVVDGAGNLQLFGSNAN.............................
BjTCP5a    PTYPSFLGAS...HHVVDGAGNLQLFSSNANRNNR.........................
BjTCP5b    PTYPSFLGAS.HHHHVVDGAGNLQLFSSNANTASQQQMMTGNSSLIRPFHHLMSSNHDTD
AtTCP5     PTYPSFLGAS.HHHHVVDGAGHLQLFSSNSNTASQQHMMPGNTSLIRPFHHLMSSNHDTD
BjTCP13a   QMPLMGSSSSS.ASQNISTLYSLLHGSSSNNGSSNRMSSVQLNQTSRGSDNPM.......
BjTCP13c   QMPLMGSSSSSSASQNISTLYSLLHGSSS....SNRMSSVQLNRTNRGSDNHM.......
BjTCP13b   QMPLMGSSSSS...QNISTLYSLLHGNSNPMPSVQFDRK.....................
AtTCP13    ............................................................
BjTCP24a   SNSHSHFLNNNN..................VNQRPISFMATPPPLDHHN.HQLPATFDGR
BjTCP24b   SNSHSLLHNNNNNN................VNQRQISFMATPPPLDHHSHQQLPATFDGR
BjTCP24c   SNSQSLFLSNNNNNNVNQRSISSSSSSSSPMDSQSISFFMAPP..LHHHTHQFPEAVDGR
BjTCP24d   SNSQPLFLNNNNN..INQRSISSSSSSSSPMDSQSISFFMSPP..LHHHSPHLPEAFDGR
AtTCP24    SNSQSLFLNNNNN.ITQRSSISSSSSSSSPMDSQSISFFMATPPPLDHHNHQLPETFDGR
BjTCP2a    SNSSNHHHHHQQQ.QQQSLLANLQRLSSSEGGGGPQFLFG..ALPAENHHHNN.....HQ
BjTCP2d    SNSSNHHQQQ.......SFLANLQRFSSSEGGGGPQFLFG..ALPAENHHPNN.....HQ
BjTCP2b    SNSTNHHHHH......QSFLANLQRFPSSEGGGGPQFLFGNGALPAENHHPHHNNNN.HQ
BjTCP2c    SNSTNHHHQH......QSFLANLQRFPSSEGGGGPQFLFG...MPAENQHPHHNNNNNHQ
AtTCP2     SNSTNQHQSF.........LANLQRFPTSESGGGPQFLFG..ALPAENHHHNH......Q
BjTCP3     SINTPMLRTWFDPYHHH....QSITTNDHHHHHPYQS.......AIPGIAFASSGELSGL
AtTCP3     SINTPMIRAWFDPHHHHHHHQQSMTTDDLHHHHPYHIPPGIHQSAIPGIAFASSGEFSGF
AtTCP4     SSYSPMIRAWFDPHHHH....QSISTDDLNHHHHLPPP..VHQSAIPGIGFASGEFSSGF
AtTCP16    .................TMTDHAPSTNYSTISDNYNSTFNGNATASDTTSAATTTATTTV
AtTCP10    QQQQRGTLQSSLFPHSFRSWDHHQTTSDHHHHQNQASSMFASSSQYGSHGMMMMQGLSFP
```

```
             280
BjTCP19    DNTSPS......................................................
AtTCP19    NSSPSSCHET..................................................
BjTCP9a    AAPRSSNH....................................................
BjTCP9c    TARSANR.....................................................
BjTCP9b    TARSSSNH....................................................
BjTCP9d    TARSSSNH....................................................
AtTCP9     TARSSNH.....................................................
AtTCP11    RRHH........................................................
BjTCP21b   ............................................................
BjTCP21d   ............................................................
BjTCP21a   ............................................................
BjTCP21e   ............................................................
BjTCP21c   ............................................................
BjTCP21f   ............................................................
AtTCP21    ............................................................
BjTCP7a    ............................................................
BjTCP7e    ............................................................
BjTCP7b    ............................................................
BjTCP7d    ............................................................
BjTCP7c    ............................................................
AtTCP7     ............................................................
BjTCP22a   QAS.ENGGDDKK................................................
BjTCP22c   QVS.ENGGDDKK................................................
AtTCP22    QAS.ENGGDDKK................................................
BjTCP22b   AEASENGGDDKK................................................
BjTCP22d   AEASENGGDDTK................................................
AtTCP23    PTIDGSP.....................................................
BjTCP8a    SRDENSNSDG..................................................
BjTCP8b    SRDENSNSDG..................................................
AtTCP8     SREENSNSSE..................................................
BjTCP15a   HSHQEDGSTSHHS...............................................
BjTCP15d   HNHQEDGSTSHHS...............................................
BjTCP15c   HSHQEDGSTSHHS...............................................
BjTCP15e   HSHQEDGSTSHHS...............................................
BjTCP15b   HNHQEDGSTSHHS...............................................
BjTCP15f   HNHQEDGSTSHHS...............................................
AtTCP15    HNHQEDGSTSHHS...............................................
BjTCP14a   YGRQVSGESQASDSLGGGEEDPQD....................................
BjTCP14b   YGRQVSGESQASDSLGGGEEDPQD....................................
AtTCP14    YGRQVSGDSQASGSLGGGDEEDQQD...................................
BjTCP20a   GSGR........................................................
BjTCP20c   GSGR........................................................
AtTCP20    GSGR........................................................
BjTCP20b   GSF.........................................................
BjTCP6     LNLWFSLLRYAYIVCEMSEIYAFGGISSLVLLGYAFCGKMCLFYSLVCLNSLSMALMYLI
AtTCP6     ............................................................
BjTCP1a    VLVQDSSLA...................................................
BjTCP1e    VWIQDYLL....................................................
BjTCP1b    V...........................................................
BjTCP1c    ............................................................
BjTCP1d    ............................................................
AtTCP1     VWIQDSFVN...................................................
BjTCP12a   FSLYDYLCY...................................................
BjTCP12b   FSLYDYLCY...................................................
AtTCP12    FSLYDYLCY...................................................
BjTCP18a   DLMYNYHNM...................................................
BjTCP18d   DLVYNYHSMC..................................................
BjTCP18b   NM..........................................................
BjTCP18c   DFMYNYHNM...................................................
AtTCP18    DLMHNYQNMY..................................................
BjTCP17a   ............................................................
BjTCP17b   ............................................................
AtTCP17    ............................................................
BjTCP5c    ............................................................
BjTCP5a    ............................................................
BjTCP5b    RHSSDDNDSDS.................................................
AtTCP5     HHSSDNESDS..................................................
BjTCP13a   ............................................................
BjTCP13c   ............................................................
BjTCP13b   ............................................................
AtTCP13    ............................................................
BjTCP24a   LYLYYGEGNRSSDDKGKDGR........................................
BjTCP24b   LYLYYGEGNRSSDDKAKDGS........................................
BjTCP24c   LYLYYGEGNRSSDDKGKERR........................................
BjTCP24d   LYLYYGEGKEKGKDQR............................................
AtTCP24    LYLYYGEGNRSSDDKAKERR........................................
BjTCP2a    FQLYYENGCRNS.DQKGKGKN.......................................
BjTCP2d    FQLYYENGCRNS.DQKGKGKN.......................................
BjTCP2b    FQLYYENGCRNS.DQKGKGKN.......................................
BjTCP2c    FQLYYENGCRNS.DQKGKGKN.......................................
AtTCP2     FQLYYENGCRNSSEHKGKGKN.......................................
BjTCP3     RVPVRYQS..EQDDHG..DNPSSASSDSRH..............................
AtTCP3     RIPARFQG..EQEEHGGDNKPSSASSDSRH..............................
AtTCP4     RIPARFQGQEEEQHDGLTHKPSSASSISRH..............................
AtTCP16    ............................................................
AtTCP10    NTTRLLHGEEATQPNSSSSPPNSHL...................................
```

```
BjTCP19   ............................................................
AtTCP19   ............................................................
BjTCP9a   ............................................................
BjTCP9c   ............................................................
BjTCP9b   ............................................................
BjTCP9d   ............................................................
AtTCP9    ............................................................
AtTCP11   ............................................................
BjTCP21b  ............................................................
BjTCP21d  ............................................................
BjTCP21a  ............................................................
BjTCP21e  ............................................................
BjTCP21c  ............................................................
BjTCP21f  ............................................................
AtTCP21   ............................................................
BjTCP7a   ............................................................
BjTCP7e   ............................................................
BjTCP7b   ............................................................
BjTCP7d   ............................................................
BjTCP7c   ............................................................
AtTCP7    ............................................................
BjTCP22a  ............................................................
BjTCP22c  ............................................................
AtTCP22   ............................................................
BjTCP22b  ............................................................
BjTCP22d  ............................................................
AtTCP23   ............................................................
BjTCP8a   ............................................................
BjTCP8b   ............................................................
AtTCP8    ............................................................
BjTCP15a  ............................................................
BjTCP15d  ............................................................
BjTCP15c  ............................................................
BjTCP15e  ............................................................
BjTCP15b  ............................................................
BjTCP15f  ............................................................
AtTCP15   ............................................................
BjTCP14a  ............................................................
BjTCP14b  ............................................................
AtTCP14   ............................................................
BjTCP20a  ............................................................
BjTCP20c  ............................................................
AtTCP20   ............................................................
BjTCP20b  ............................................................
BjTCP6    TLSYVGFAPKCTELRIEILSYRVLGVKISLVGFILFERVCLEAELLNESLLFFFFVFCRP
AtTCP6    ............................................................
BjTCP1a   ............................................................
BjTCP1e   ............................................................
BjTCP1b   ............................................................
BjTCP1c   ............................................................
BjTCP1d   ............................................................
AtTCP1    ............................................................
BjTCP12a  ............................................................
BjTCP12b  ............................................................
AtTCP12   ............................................................
BjTCP18a  ............................................................
BjTCP18d  ............................................................
BjTCP18b  ............................................................
BjTCP18c  ............................................................
AtTCP18   ............................................................
BjTCP17a  ............................................................
BjTCP17b  ............................................................
AtTCP17   ............................................................
BjTCP5c   ............................................................
BjTCP5a   ............................................................
BjTCP5b   ............................................................
AtTCP5    ............................................................
BjTCP13a  ............................................................
BjTCP13c  ............................................................
BjTCP13b  ............................................................
AtTCP13   ............................................................
BjTCP24a  ............................................................
BjTCP24b  ............................................................
BjTCP24c  ............................................................
BjTCP24d  ............................................................
AtTCP24   ............................................................
BjTCP2a   ............................................................
BjTCP2d   ............................................................
BjTCP2b   ............................................................
BjTCP2c   ............................................................
AtTCP2    ............................................................
BjTCP3    ............................................................
AtTCP3    ............................................................
AtTCP4    ............................................................
AtTCP16   ............................................................
AtTCP10   ............................................................
```

```
BjTCP19    ............................................................
AtTCP19    ............................................................
BjTCP9a    ............................................................
BjTCP9c    ............................................................
BjTCP9b    ............................................................
BjTCP9d    ............................................................
AtTCP9     ............................................................
AtTCP11    ............................................................
BjTCP21b   ............................................................
BjTCP21d   ............................................................
BjTCP21a   ............................................................
BjTCP21e   ............................................................
BjTCP21c   ............................................................
BjTCP21f   ............................................................
AtTCP21    ............................................................
BjTCP7a    ............................................................
BjTCP7e    ............................................................
BjTCP7b    ............................................................
BjTCP7d    ............................................................
BjTCP7c    ............................................................
AtTCP7     ............................................................
BjTCP22a   ............................................................
BjTCP22c   ............................................................
AtTCP22    ............................................................
BjTCP22b   ............................................................
BjTCP22d   ............................................................
AtTCP23    ............................................................
BjTCP8a    ............................................................
BjTCP8b    ............................................................
AtTCP8     ............................................................
BjTCP15a   ............................................................
BjTCP15d   ............................................................
BjTCP15c   ............................................................
BjTCP15e   ............................................................
BjTCP15b   ............................................................
BjTCP15f   ............................................................
AtTCP15    ............................................................
BjTCP14a   ............................................................
BjTCP14b   ............................................................
AtTCP14    ............................................................
BjTCP20a   ............................................................
BjTCP20c   ............................................................
AtTCP20    ............................................................
BjTCP20b   ............................................................
BjTCP6     QTMKWPSNAIANYKCENIITLNSPLNIKSEFKIIYYPLALGVCFGDLITQLTESSTFTCI
AtTCP6     ............................................................
BjTCP1a    ............................................................
BjTCP1e    ............................................................
BjTCP1b    ............................................................
BjTCP1c    ............................................................
BjTCP1d    ............................................................
AtTCP1     ............................................................
BjTCP12a   ............................................................
BjTCP12b   ............................................................
AtTCP12    ............................................................
BjTCP18a   ............................................................
BjTCP18d   ............................................................
BjTCP18b   ............................................................
BjTCP18c   ............................................................
AtTCP18    ............................................................
BjTCP17a   ............................................................
BjTCP17b   ............................................................
AtTCP17    ............................................................
BjTCP5c    ............................................................
BjTCP5a    ............................................................
BjTCP5b    ............................................................
AtTCP5     ............................................................
BjTCP13a   ............................................................
BjTCP13c   ............................................................
BjTCP13b   ............................................................
AtTCP13    ............................................................
BjTCP24a   ............................................................
BjTCP24b   ............................................................
BjTCP24c   ............................................................
BjTCP24d   ............................................................
AtTCP24    ............................................................
BjTCP2a    ............................................................
BjTCP2d    ............................................................
BjTCP2b    ............................................................
BjTCP2c    ............................................................
AtTCP2     ............................................................
BjTCP3     ............................................................
AtTCP3     ............................................................
AtTCP4     ............................................................
AtTCP16    ............................................................
AtTCP10    ............................................................
```

```
BjTCP19    ............................................................
AtTCP19    ............................................................
BjTCP9a    ............................................................
BjTCP9c    ............................................................
BjTCP9b    ............................................................
BjTCP9d    ............................................................
AtTCP9     ............................................................
AtTCP11    ............................................................
BjTCP21b   ............................................................
BjTCP21d   ............................................................
BjTCP21a   ............................................................
BjTCP21e   ............................................................
BjTCP21c   ............................................................
BjTCP21f   ............................................................
AtTCP21    ............................................................
BjTCP7a    ............................................................
BjTCP7e    ............................................................
BjTCP7b    ............................................................
BjTCP7d    ............................................................
BjTCP7c    ............................................................
AtTCP7     ............................................................
BjTCP22a   ............................................................
BjTCP22c   ............................................................
AtTCP22    ............................................................
BjTCP22b   ............................................................
BjTCP22d   ............................................................
AtTCP23    ............................................................
BjTCP8a    ............................................................
BjTCP8b    ............................................................
AtTCP8     ............................................................
BjTCP15a   ............................................................
BjTCP15d   ............................................................
BjTCP15c   ............................................................
BjTCP15e   ............................................................
BjTCP15b   ............................................................
BjTCP15f   ............................................................
AtTCP15    ............................................................
BjTCP14a   ............................................................
BjTCP14b   ............................................................
AtTCP14    ............................................................
BjTCP20a   ............................................................
BjTCP20c   ............................................................
AtTCP20    ............................................................
BjTCP20b   ............................................................
BjTCP6     VCLSFDLSGYICGRTSFAALWDKSAVNTLASITRKLQLRKLVNNEYVGENSESLLGNSCT
AtTCP6     ............................................................
BjTCP1a    ............................................................
BjTCP1e    ............................................................
BjTCP1b    ............................................................
BjTCP1c    ............................................................
BjTCP1d    ............................................................
AtTCP1     ............................................................
BjTCP12a   ............................................................
BjTCP12b   ............................................................
AtTCP12    ............................................................
BjTCP18a   ............................................................
BjTCP18d   ............................................................
BjTCP18b   ............................................................
BjTCP18c   ............................................................
AtTCP18    ............................................................
BjTCP17a   ............................................................
BjTCP17b   ............................................................
AtTCP17    ............................................................
BjTCP5c    ............................................................
BjTCP5a    ............................................................
BjTCP5b    ............................................................
AtTCP5     ............................................................
BjTCP13a   ............................................................
BjTCP13c   ............................................................
BjTCP13b   ............................................................
AtTCP13    ............................................................
BjTCP24a   ............................................................
BjTCP24b   ............................................................
BjTCP24c   ............................................................
BjTCP24d   ............................................................
AtTCP24    ............................................................
BjTCP2a    ............................................................
BjTCP2d    ............................................................
BjTCP2b    ............................................................
BjTCP2c    ............................................................
AtTCP2     ............................................................
BjTCP3     ............................................................
AtTCP3     ............................................................
AtTCP4     ............................................................
AtTCP16    ............................................................
AtTCP10    ............................................................
```

```
BjTCP19    ............................................................
AtTCP19    ............................................................
BjTCP9a    ............................................................
BjTCP9c    ............................................................
BjTCP9b    ............................................................
BjTCP9d    ............................................................
AtTCP9     ............................................................
AtTCP11    ............................................................
BjTCP21b   ............................................................
BjTCP21d   ............................................................
BjTCP21a   ............................................................
BjTCP21e   ............................................................
BjTCP21c   ............................................................
BjTCP21f   ............................................................
AtTCP21    ............................................................
BjTCP7a    ............................................................
BjTCP7e    ............................................................
BjTCP7b    ............................................................
BjTCP7d    ............................................................
BjTCP7c    ............................................................
AtTCP7     ............................................................
BjTCP22a   ............................................................
BjTCP22c   ............................................................
AtTCP22    ............................................................
BjTCP22b   ............................................................
BjTCP22d   ............................................................
AtTCP23    ............................................................
BjTCP8a    ............................................................
BjTCP8b    ............................................................
AtTCP8     ............................................................
BjTCP15a   ............................................................
BjTCP15d   ............................................................
BjTCP15c   ............................................................
BjTCP15e   ............................................................
BjTCP15b   ............................................................
BjTCP15f   ............................................................
AtTCP15    ............................................................
BjTCP14a   ............................................................
BjTCP14b   ............................................................
AtTCP14    ............................................................
BjTCP20a   ............................................................
BjTCP20c   ............................................................
AtTCP20    ............................................................
BjTCP20b   ............................................................
BjTCP6     TFEALSAFVWRARTKSLKMLNDQKTKLLFAVDGRAKFEPPLPKGYFGNGIVLTNSICEAG
AtTCP6     ............................................................
BjTCP1a    ............................................................
BjTCP1e    ............................................................
BjTCP1b    ............................................................
BjTCP1c    ............................................................
BjTCP1d    ............................................................
AtTCP1     ............................................................
BjTCP12a   ............................................................
BjTCP12b   ............................................................
AtTCP12    ............................................................
BjTCP18a   ............................................................
BjTCP18d   ............................................................
BjTCP18b   ............................................................
BjTCP18c   ............................................................
AtTCP18    ............................................................
BjTCP17a   ............................................................
BjTCP17b   ............................................................
AtTCP17    ............................................................
BjTCP5c    ............................................................
BjTCP5a    ............................................................
BjTCP5b    ............................................................
AtTCP5     ............................................................
BjTCP13a   ............................................................
BjTCP13c   ............................................................
BjTCP13b   ............................................................
AtTCP13    ............................................................
BjTCP24a   ............................................................
BjTCP24b   ............................................................
BjTCP24c   ............................................................
BjTCP24d   ............................................................
AtTCP24    ............................................................
BjTCP2a    ............................................................
BjTCP2d    ............................................................
BjTCP2b    ............................................................
BjTCP2c    ............................................................
AtTCP2     ............................................................
BjTCP3     ............................................................
AtTCP3     ............................................................
AtTCP4     ............................................................
AtTCP16    ............................................................
AtTCP10    ............................................................
```

```
BjTCP19    ............................................................
AtTCP19    ............................................................
BjTCP9a    ............................................................
BjTCP9c    ............................................................
BjTCP9b    ............................................................
BjTCP9d    ............................................................
AtTCP9     ............................................................
AtTCP11    ............................................................
BjTCP21b   ............................................................
BjTCP21d   ............................................................
BjTCP21a   ............................................................
BjTCP21e   ............................................................
BjTCP21c   ............................................................
BjTCP21f   ............................................................
AtTCP21    ............................................................
BjTCP7a    ............................................................
BjTCP7e    ............................................................
BjTCP7b    ............................................................
BjTCP7d    ............................................................
BjTCP7c    ............................................................
AtTCP7     ............................................................
BjTCP22a   ............................................................
BjTCP22c   ............................................................
AtTCP22    ............................................................
BjTCP22b   ............................................................
BjTCP22d   ............................................................
AtTCP23    ............................................................
BjTCP8a    ............................................................
BjTCP8b    ............................................................
AtTCP8     ............................................................
BjTCP15a   ............................................................
BjTCP15d   ............................................................
BjTCP15c   ............................................................
BjTCP15e   ............................................................
BjTCP15b   ............................................................
BjTCP15f   ............................................................
AtTCP15    ............................................................
BjTCP14a   ............................................................
BjTCP14b   ............................................................
AtTCP14    ............................................................
BjTCP20a   ............................................................
BjTCP20c   ............................................................
AtTCP20    ............................................................
BjTCP20b   ............................................................
BjTCP6     ELTEKPLSFAVGLVREAIKMVTDGYMRSAIDYFEVTRARPSLSSTLLITTWSRLGFHTTD
AtTCP6     ............................................................
BjTCP1a    ............................................................
BjTCP1e    ............................................................
BjTCP1b    ............................................................
BjTCP1c    ............................................................
BjTCP1d    ............................................................
AtTCP1     ............................................................
BjTCP12a   ............................................................
BjTCP12b   ............................................................
AtTCP12    ............................................................
BjTCP18a   ............................................................
BjTCP18d   ............................................................
BjTCP18b   ............................................................
BjTCP18c   ............................................................
AtTCP18    ............................................................
BjTCP17a   ............................................................
BjTCP17b   ............................................................
AtTCP17    ............................................................
BjTCP5c    ............................................................
BjTCP5a    ............................................................
BjTCP5b    ............................................................
AtTCP5     ............................................................
BjTCP13a   ............................................................
BjTCP13c   ............................................................
BjTCP13b   ............................................................
AtTCP13    ............................................................
BjTCP24a   ............................................................
BjTCP24b   ............................................................
BjTCP24c   ............................................................
BjTCP24d   ............................................................
AtTCP24    ............................................................
BjTCP2a    ............................................................
BjTCP2d    ............................................................
BjTCP2b    ............................................................
BjTCP2c    ............................................................
AtTCP2     ............................................................
BjTCP3     ............................................................
AtTCP3     ............................................................
AtTCP4     ............................................................
AtTCP16    ............................................................
AtTCP10    ............................................................
```

```
BjTCP19    .....................................................
AtTCP19    .....................................................
BjTCP9a    .....................................................
BjTCP9c    .....................................................
BjTCP9b    .....................................................
BjTCP9d    .....................................................
AtTCP9     .....................................................
AtTCP11    .....................................................
BjTCP21b   .....................................................
BjTCP21d   .....................................................
BjTCP21a   .....................................................
BjTCP21e   .....................................................
BjTCP21c   .....................................................
BjTCP21f   .....................................................
AtTCP21    .....................................................
BjTCP7a    .....................................................
BjTCP7e    .....................................................
BjTCP7b    .....................................................
BjTCP7d    .....................................................
BjTCP7c    .....................................................
AtTCP7     .....................................................
BjTCP22a   .....................................................
BjTCP22c   .....................................................
AtTCP22    .....................................................
BjTCP22b   .....................................................
BjTCP22d   .....................................................
AtTCP23    .....................................................
BjTCP8a    .....................................................
BjTCP8b    .....................................................
AtTCP8     .....................................................
BjTCP15a   .....................................................
BjTCP15d   .....................................................
BjTCP15c   .....................................................
BjTCP15e   .....................................................
BjTCP15b   .....................................................
BjTCP15f   .....................................................
AtTCP15    .....................................................
BjTCP14a   .....................................................
BjTCP14b   .....................................................
AtTCP14    .....................................................
BjTCP20a   .....................................................
BjTCP20c   .....................................................
AtTCP20    .....................................................
BjTCP20b   .....................................................
BjTCP6     FGWGEPVLSGPVALPEKEVTLFLSHGEERRSINVLLGLPASAMDVFQELFLQI
AtTCP6     .....................................................
BjTCP1a    .....................................................
BjTCP1e    .....................................................
BjTCP1b    .....................................................
BjTCP1c    .....................................................
BjTCP1d    .....................................................
AtTCP1     .....................................................
BjTCP12a   .....................................................
BjTCP12b   .....................................................
AtTCP12    .....................................................
BjTCP18a   .....................................................
BjTCP18d   .....................................................
BjTCP18b   .....................................................
BjTCP18c   .....................................................
AtTCP18    .....................................................
BjTCP17a   .....................................................
BjTCP17b   .....................................................
AtTCP17    .....................................................
BjTCP5c    .....................................................
BjTCP5a    .....................................................
BjTCP5b    .....................................................
AtTCP5     .....................................................
BjTCP13a   .....................................................
BjTCP13c   .....................................................
BjTCP13b   .....................................................
AtTCP13    .....................................................
BjTCP24a   .....................................................
BjTCP24b   .....................................................
BjTCP24c   .....................................................
BjTCP24d   .....................................................
AtTCP24    .....................................................
BjTCP2a    .....................................................
BjTCP2d    .....................................................
BjTCP2b    .....................................................
BjTCP2c    .....................................................
AtTCP2     .....................................................
BjTCP3     .....................................................
AtTCP3     .....................................................
AtTCP4     .....................................................
AtTCP16    .....................................................
AtTCP10    .....................................................
```
