# Supplementary figures and images for: Genome-wide identification and characterization of TCP family genes in Brassica juncea var. tumida
Source: PeerJ. 2020 May 14;8:e9130. doi: 10.7717/peerj.9130 (PMC7231505; doi:10.7717/peerj.9130)

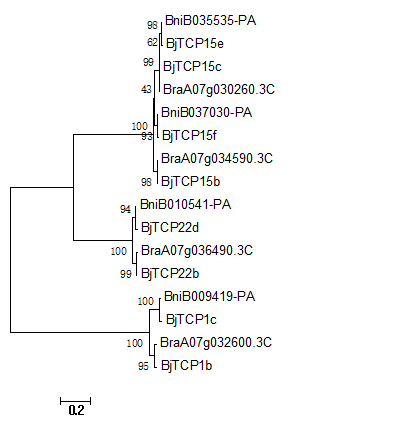

Supplement: Figure S2 [file peerj-08-9130-s002.tif]

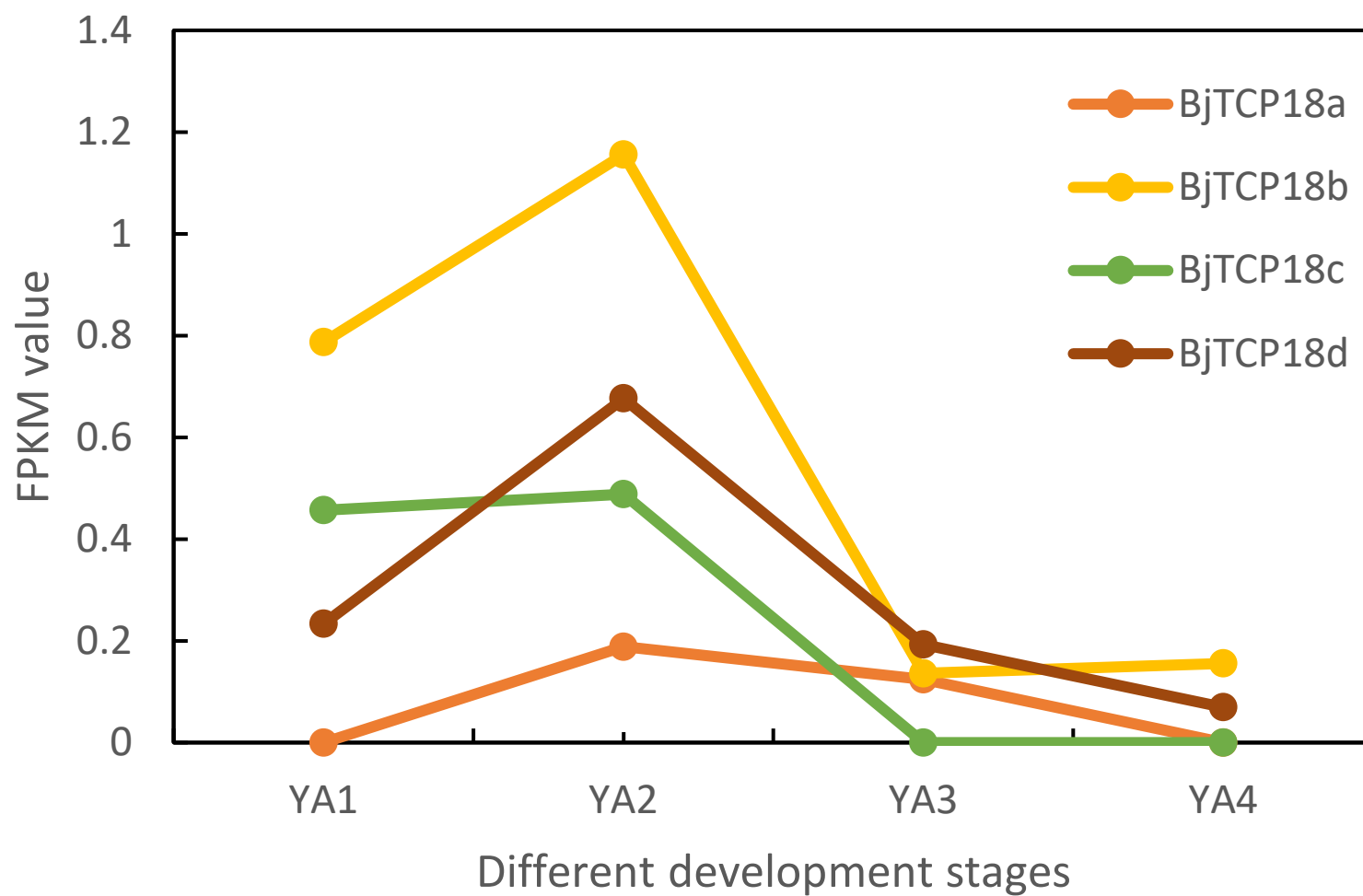

Supplement: Figure S3 [file peerj-08-9130-s003.pdf]
